# ChAHP2 and ChAHP control diverse retrotransposons by complementary activities

**DOI:** 10.1101/gad.351769.124

**Published:** 2024-06-01

**Authors:** Josip Ahel, Aparna Pandey, Michaela Schwaiger, Fabio Mohn, Anja Basters, Georg Kempf, Aude Andriollo, Lucas Kaaij, Daniel Hess, Marc Bühler

**Affiliations:** 1 Friedrich Miescher Institute for Biomedical Research, Basel 4056, Switzerland;; 2 Swiss Institute of Bioinformatics, Basel 4056, Switzerland;; 3 University of Basel, Basel 4003, Switzerland

**Keywords:** ADNP, ADNP2, CHD4, ChAHP, ChAHP2, HP1, retrotransposon

## Abstract

Retrotransposon control in mammals is an intricate process that is effectuated by a broad network of chromatin regulatory pathways. We previously discovered ChAHP, a protein complex with repressive activity against short interspersed element (SINE) retrotransposons that is composed of the transcription factor ADNP, chromatin remodeler CHD4, and HP1 proteins. Here we identify ChAHP2, a protein complex homologous to ChAHP, in which ADNP is replaced by ADNP2. ChAHP2 is predominantly targeted to endogenous retroviruses (ERVs) and long interspersed elements (LINEs) via HP1β-mediated binding of H3K9 trimethylated histones. We further demonstrate that ChAHP also binds these elements in a manner mechanistically equivalent to that of ChAHP2 and distinct from DNA sequence-specific recruitment at SINEs. Genetic ablation of ADNP2 alleviates ERV and LINE1 repression, which is synthetically exacerbated by additional depletion of ADNP. Together, our results reveal that the ChAHP and ChAHP2 complexes function to control both nonautonomous and autonomous retrotransposons by complementary activities, further adding to the complexity of mammalian transposon control.

## Abstract (precis)

In this study, Ahel et al. describe a novel protein complex, ChAHP2, involved in transposon silencing, that is predominantly targeted to ERVs and LINEs via HP1β-mediated binding of H3K9 trimethylated histones. Analogous to but distinct from the SINE-repressive ChAHP complex, the ChAHP2 complex suppresses ERVs and LINE1 elements, with both complexes functioning complementarily to bring about efficient retrotransposon repression.

Retrotransposons make up a substantial proportion of mammalian genomes and present a potential threat due to their ability to amplify and insert into new genomic loci. The complement of retrotransposons is diverse in terms of both sequence and origin, making their regulation mechanistically challenging (Kazazian 2004; Platt et al. 2018). To overcome this diversity, cells use a vast number of different specificity factors that guide a variety of interlinked chromatin-modulating activities. One of the best-described examples of this is the repression of endogenous retroviruses (ERVs). Here, the long terminal repeat (LTR) region of the ERV, which normally acts as a promoter, is recognized by sequence-specific transcription factors (TFs) from the Krüppel-associated box zinc finger (KRAB-ZFP) protein family (Wolf and Goff 2009; Ecco et al. 2017; Imbeault et al. 2017). These TFs then recruit the corepressor TRIM28, which in turn interacts with SETDB1, guiding the deposition of H3K9me3 and establishing a transcriptionally silent state (Schultz et al. 2002; Wolf and Goff 2009; Matsui et al. 2010; Rowe et al. 2010; Quenneville et al. 2012). This process is potentiated by HP1 proteins, which interact with both TRIM28 and H3K9me3 (Lechner et al. 2000; Ayyanathan et al. 2003) and assist with recruitment of additional chromatin regulators including histone deacetylases (Schultz et al. 2001) and demethylases (Macfarlan et al. 2011), DNA methyltransferases (Smallwood et al. 2007), and chromatin remodelers such as ATRX–DAXX, the NuRD complex, MORC3, and SMARCAD1 (Schultz et al. 2001; Sadic et al. 2015; Sachs et al. 2019; Groh et al. 2021). Ultimately, this leads to removal of activating histone acetylation and methylation marks, methylation of the underlying DNA, and remodeling of the local chromatin structure, which altogether confer transcriptional repression to the targeted loci (Rowe et al. 2010; Quenneville et al. 2012; Turelli et al. 2014; Wang et al. 2022).

Additional important repressive activity is provided by the HUSH complex, which is targeted to non-LTR-containing long interspersed elements (LINEs) such as LINE1 and a subset of ERVs (Tchasovnikarova et al. 2015; Liu et al. 2018; Robbez-Masson et al. 2018). This targeting occurs via a non-DNA sequence-specific mechanism based on the absence of introns, length of the transcript, and high adenine content (Seczynska et al. 2022). Similar to KRAB-ZFP-mediated repression, HUSH represses its targets via SETDB1-dependent deposition of H3K9me3 and chromatin remodeling (Tchasovnikarova et al. 2015; Liu et al. 2018; Robbez-Masson et al. 2018; Müller et al. 2021).

This multitude of transposon recognition and silencing pathways is essential because novel insertions of retrotransposons can induce mutations causing a variety of heritable and somatic diseases (Teugels et al. 2005; Hancks and Kazazian 2016; Gonçalves et al. 2017; Qian et al. 2017; Aneichyk et al. 2018). Both ERVs and LINE1 retrotransposons are autonomous, encoding all components necessary for their own retrotransposition (Wicker et al. 2007; Platt et al. 2018). Notably, the LINE1 retrotransposition machinery can additionally support the transposition of the nonautonomous short interspersed nuclear elements (SINEs) (Dewannieux et al. 2003; Dewannieux and Heidmann 2005; Raiz et al. 2012). These elements are often derived from tRNAs or 7SL RNAs, and their regulation is less well understood (Ullu and Tschudi 1984; Daniels and Deininger 1985; Dewannieux et al. 2003; Dewannieux and Heidmann 2005; Raiz et al. 2012; Varshney et al. 2015). We recently discovered that they are partly repressed by the ChAHP complex, which unites the transcription factor ADNP, chromatin remodeler CHD4, and HP1 proteins into a stably associated module (Ostapcuk et al. 2018; Kaaij et al. 2019). Genetic removal of ADNP allows increased expression of evolutionarily less divergent SINE B2 elements in mouse embryonic stem cells (mESCs), accompanied by an increase in chromatin accessibility and CTCF binding (Ostapcuk et al. 2018; Kaaij et al. 2019). Phenotypically, defects in in vitro differentiation and mouse development have been observed upon *adnp* knockout, resulting in a penetrant embryonic lethality phenotype by embryonic day 9.5 (E9.5) (Mandel et al. 2007; Ostapcuk et al. 2018). In humans, even heterozygous partial truncations of ADNP that abrogate interactions with HP1 proteins result in a severe autism spectrum syndrome typified by developmental defects, compromised function of multiple organ systems, and intellectual disability (Helsmoortel et al. 2014). Although their underlying molecular deficiency is unclear, these striking phenotypes highlight the importance of identifying hitherto unknown retrotransposon-associated chromatin regulators.

Here we report the discovery of ChAHP2, a protein complex with composition similar to that of ChAHP. ChAHP2 is defined by ADNP2, a paralog of ADNP widely present in vertebrates (Altenhoff et al. 2019). ChAHP2 chromatin binding specificity is distinct from ChAHP, predominantly associating with ERV and LINE1 retrotransposons via HP1β-mediated binding of H3K9 trimethylated histones. Through complementary activities, the ChAHP and ChAHP2 complexes control a wide variety of molecularly disparate retrotransposons, including SINEs, LINEs, and ERVs.

## Results

### ADNP2 interacts with HP1β and CHD4 to form a distinct ChAHP2 complex

**Figure 1.**
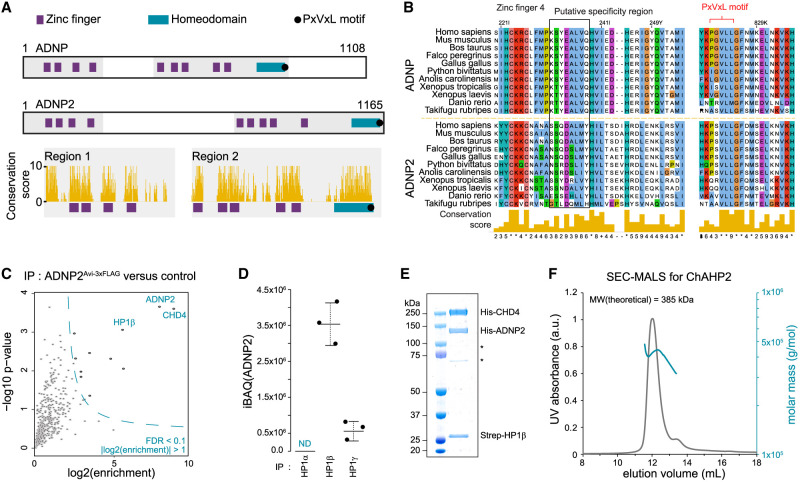
ADNP2 interacts with HP1β and CHD4 to form a ChAHP2 complex. (*A*) Protein architecture schematics of ADNP and ADNP2, drawn to scale. The amino acid conservation score (Clustal/JalView) is given for the highlighted regions. (*B*) Predicted ADNP or ADNP2 ortholog sequences from species representing different vertebrate classes were aligned using Clustal Ω (Madeira et al. 2022), ordered by species, and visualized with JalView (Waterhouse et al. 2009). The conservation scores were scaled and are used in *A*. Alignment excerpts for the regions containing zinc finger 4 (counting from the N terminus) and the PxVxL motif are displayed. The putative region responsible for sequence specificity of the zinc finger is highlighted. Alignment excerpts covering all other zinc fingers are shown in Supplemental Figure S1A. (*C*) Cells expressing endogenously edited ADNP2^Avi-3xFLAG^ or parental untagged cells were subjected to immunoprecipitation with streptavidin and analyzed by proteomics (*n* = 3). The dashed line represents the significance cutoff at FDR = 0.1 and log_2_Enrichment > 1. (*D*) Abundance of proteins from the HP1 proteins recovered in the experiment described in *C*, expressed as iBAQ values. (*E*,*F*) His-ADNP2 and Strep-HP1β were coexpressed and His-CHD4 was expressed separately using baculovirus transductions in insect cells before lysing the mixed cell pools and pull-down against Strep, followed by electrophoresis and Coomassie staining (*E*) and SEC-MALS (*F*).

The domain architecture of ADNP2 resembles that of ADNP, with nine zinc fingers distributed in two N-terminal clusters and one C-terminal homeodomain. These domains are the only regions showing overall high conservation, while the putatively poorly structured linker region between the zinc finger clusters as well as the C-terminal unstructured region of ADNP are not well conserved between the two paralogs (Fig. 1A). Notably, although the zinc-coordinating residues and overall predicted fold of the zinc fingers are conserved, the residues that normally determine sequence specificity of zinc fingers vary between ADNP and ADNP2 (Fig. 1B; Supplemental Fig. S1A). In contrast, the C-terminal HP1 interaction motif (PxVxL) (Thiru et al. 2004; Mosch et al. 2011) is well conserved. Moreover, ADNP2 is copurified in CHD4 immunoprecipitations (IPs) (Ostapcuk et al. 2018), suggesting that the regions critical for both CHD4 and HP1β/γ interactions are conserved between the two paralogs. These observations prompted us to hypothesize that ADNP2 can form an alternative ChAHP complex with potentially different properties. To test this, we introduced an affinity tag consisting of Avi-3xFLAG to the C terminus of ADNP2 in mouse ES cells (Supplemental Fig. S1B). We then used these cells to perform affinity purifications with streptavidin and analyzed the samples by liquid chromatography-mass spectrometry (LC-MS), with the parental cell line serving as a negative control. The bait protein (ADNP2) was strongly enriched in the ADNP2 IPs compared with control, copurifying CHD4 and HP1β as the top two significantly enriched interactors (Fig. 1C). Several other proteins were identified as significantly enriched, but their enrichment was overall lower (Supplemental Table S1). This group included abundant proteins that commonly copurify in IPs of DNA binding factors, such as PARP1, hinting that these may be experimental artifacts (Mellacheruvu et al. 2013). Overall, this experiment shows that ADNP2 interacts with both HP1β and CHD4 in mouse ES cells. Such an interaction pattern is reminiscent of the ChAHP complex, with the notable difference that ADNP2 preferentially binds HP1β over HP1γ (Fig. 1D; Ostapcuk et al. 2018). Importantly, no ADNP was copurified with ADNP2 and vice versa, suggesting that their interaction networks are separate. To further probe the biochemical independence of ADNP and ADNP2, we evaluated whether the composition of either complex is affected by removal of the other. To that end, we knocked out *adnp2* from endogenously edited cells expressing ADNP^FKBP-3xFLAG-Avi^ (Supplemental Fig. S1C) and knocked out *adnp* from cells expressing ADNP2^Avi-3xFLAG^. We screened for successful gene deletion by PCR and confirmed loss of mRNA by either exon-spanning RT-qPCR or RNA sequencing (Supplemental Fig. S1D,E). No significant difference in core subunit pull-down efficiencies was apparent between the WT and KO conditions for either ADNP or ADNP2 (Supplemental Fig. S2). This indicates that competition between ADNP and ADNP2 for subunit binding does not have a major effect on complex assembly.

Finally, to directly confirm that ADNP2, HP1β, and CHD4 form a stable complex, we performed in vitro reconstitutions. First, we coexpressed His-ADNP2 and Strep-HP1β in insect cells and expressed His-CHD4 in separately transduced cells before mixing the two cell pools for lysis and streptactin pull-down. We analyzed these pull-downs using size exclusion chromatography with multiangle light scattering (SEC-MALS) and Coomassie staining. All three proteins were detected in one elution peak, with an estimated molecular weight consistent with an ADNP2–CHD4–HP1β complex (Fig. 1E,F). Together, these data demonstrate that ADNP2, CHD4, and HP1β form an independent bona fide complex, which we refer to as ChAHP2.

### ChAHP2 binds retrotransposons

**Figure 2.**
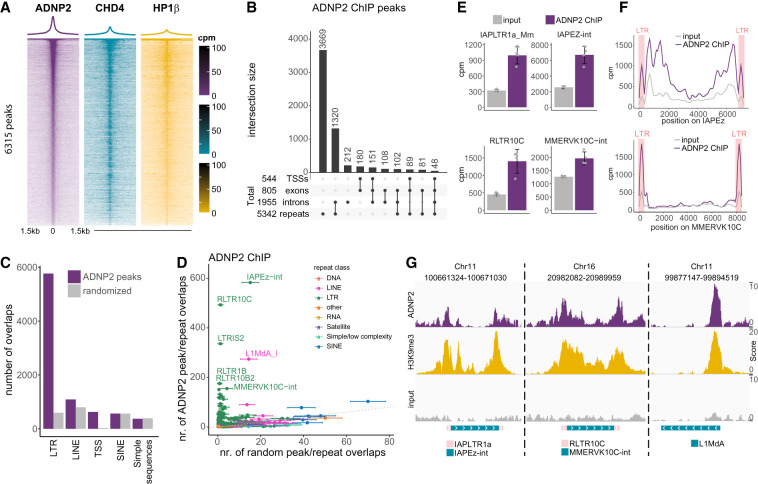
ChAHP2 predominantly binds to ERVs and LINEs. (*A*) Waterfall plots of ChIP-seq counts normalized to library size centered on summits of ADNP2 peaks (mean; *n* = 4). (*B*) Upset plot of overlaps between ADNP2 peaks and select genomic features (TSSs, exons, introns, and repeats). (*C*) Overlap numbers between repeat annotations and ADNP2 peaks or a randomized peak set of equal properties (bootstrapped 100 times; mean ± SD). (Simple sequences) Simple repeats and low-complexity regions. (*D*) Same as *C* but further split by repeat family. (*E*) Summed reads over repeat annotations normalized to library size for input and ADNP2 ChIP-seq (mean ± SD; *n* = 4). (*F*) Consensus mapping traces over repeat annotations normalized to library size for input and ADNP2 ChIP-seq (mean ± SD; *n* = 4). For IAPEz, the IAPEz-int consensus sequence was stitched with IAPLTR1a_Mm at either end, while for MMERVK10C, MMERVK10C-int was stitched with RLTR10C. The position of the stitched LTRs is highlighted. (*G*) IGV genome browser shots of select regions bound by ADNP2.

We next performed ChIP sequencing (ChIp-seq) to characterize the chromatin localization of ADNP2. Peak calling revealed 6315 regions with significant ADNP2 enrichment over input chromatin (Supplemental Table S2). These regions also showed enriched binding of CHD4 and HP1β in previously published data sets, suggesting that ChAHP2 complexes occupy these sites (Fig. 2A). Intriguingly, the majority of ADNP2 peaks were found in repeat regions, while few overlapped transcription start sites (TSSs) (Fig. 2B). Retrotransposons belonging to LTR-containing families, including several classes of ERV elements, were particularly overrepresented when compared with a randomized peak set of equal properties (Fig. 2C,D; Supplemental Fig. S3A; Supplemental Table S3). In addition, one subfamily of LINE1 elements was overrepresented (Fig. 2D; Supplemental Fig. S3A). We further supplemented these analyses with a peak-agnostic, repeat family-based approach. This agreed well with the initial analysis, showing enrichment over input at the repeat classes identified by peak calling, with both the internal sequence of ERVs and their associated LTR sequences exhibiting enrichment in ADNP2 ChIP signal (Fig. 2E). Finally, we additionally determined ADNP2 ChIP signal distribution along repeat elements using mapping to repeat consensus sequences, which showed results similar to those of the other analyses (Fig. 2F; Supplemental Fig. S3C). Together, these data indicate that ADNP2 binds several classes of retrotransposons both internally and at their terminal sequences.

### ChAHP2 is recruited to chromatin via HP1 binding to H3K9me3

**Figure 3.**
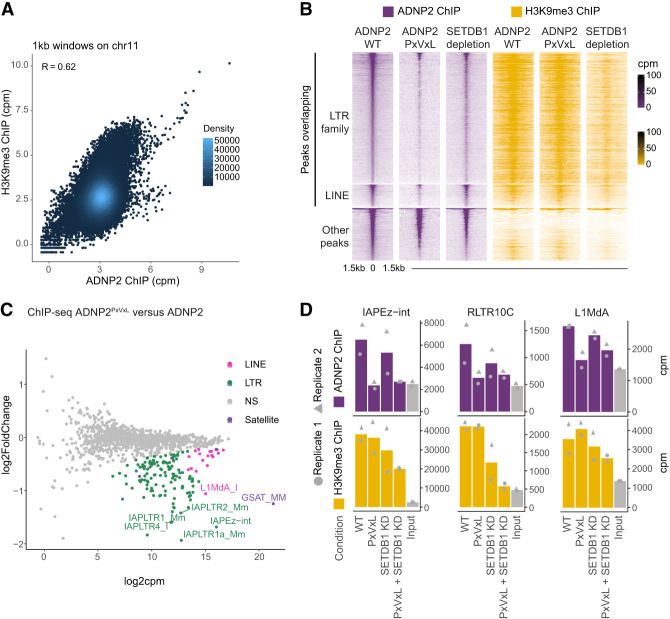
ChAHP2 binds heterochromatin in an HP1- and H3K9me3-dependent manner. (*A*) Comparison between ADNP2 and H3K9me3 ChIP-seq signal in 1 kb genomic bins across chromosome 11. (*B*) Cells expressing WT or PxVxL mutated ADNP2^Avi-3xFLAG^ before or after SETDB1 depletion were analyzed by ChIP-seq. Average ChIP counts per million (cpm) is displayed (*n* = 2). (*C*) Differential binding analysis of summed reads over repeat annotations normalized to human spike-ins comparing ADNP2^PxVxL^ ChIP and WT controls (*n* = 2). (*D*) Summed reads over repeat annotations normalized to human spike-ins for input and a series of ChIP samples as indicated (mean; *n* = 2; replicates are annotated). ADNP2 and H3K9me3 ChIPs were done simultaneously and from the same material.

Unlike many TFs that bind readily identifiable DNA sequence motifs, no motifs accounted for >15% of ADNP2-bound sites (Supplemental Fig. S3D). In addition, these motifs were not centrally enriched within ADNP2 peaks, hinting that they are not a direct specificity determinant (Supplemental Fig. S3D). Notably, a common feature of ADNP2-bound retrotransposons is the presence of H3K9 trimethylation (Fig. 2G; Matsui et al. 2010; Karimi et al. 2011; Castro-Diaz et al. 2014). Since HP1 proteins are known to bind this histone modification (Bannister et al. 2001; Lachner et al. 2001), we hypothesized that ChAHP2 is recruited to its targets by HP1β-mediated binding to H3K9me3 histone tails. Indeed, ADNP2 chromatin binding correlates well with H3K9me3 levels genome-wide (Fig. 3A). To directly test whether HP1 is required for ChAHP2 binding to chromatin, we specifically abrogated the ADNP2–HP1β interaction by introducing point mutations in the PxVxL motif of ADNP2. We verified successful editing and homozygosity by Sanger sequencing (Supplemental Fig. S4A). As expected, wild-type ADNP2 was able to pull down HP1β and CHD4, whereas ADNP2^PxVxL^ mutants were unable to bind HP1β above background levels (Supplemental Fig. S4B). Importantly, CHD4 binding was unaffected, confirming that the mutations specifically disrupt the ADNP2–HP1β interaction (Supplemental Fig. S4B). Next, we performed ChIP-seq for ADNP2 and ADNP2^PxVxL^. Mutations in the PxVxL motif resulted in a near-complete loss of ChAHP2 binding to H3K9me3-modified target regions, while H3K9me3 levels remained unchanged (Fig. 3B). These regions almost exclusively overlap LTR or LINE1 elements. Statistical analysis of significantly changing peaks corroborated this initial impression (Supplemental Fig. S4C,D). In turn, the peaks with increased binding were promoter-associated, were devoid of H3K9me3, and exhibited higher chromatin accessibility (Supplemental Fig. S4E,F). This behavior would be consistent with a redistribution of ChAHP2 from heterochromatin to euchromatin when HP1 is removed from the complex. Finally, regions without a significant change exhibited an intermediate accessibility and H3K9me3 profile (Supplemental Fig. S4F). Despite not being identified as statistically significant, ADNP2 binding was still overall reduced in this group (Supplemental Fig. S4G). These observations provide evidence that chromatin binding at the majority of ChAHP2 targets is dependent on HP1.

We next sought to test whether ChAHP2 binding depends on the presence of H3K9me3. To do this, we introduced a 2xHA-FKBP12^F36V^ degron tag at the N terminus of endogenous SETDB1, the methyltransferase responsible for depositing H3K9me3 at LTR elements (Supplemental Fig. S5A; Matsui et al. 2010; Nabet et al. 2018), and confirmed the efficacy of SETDB1 depletion by Western blotting (Supplemental Fig. S5B). We then generated ADNP2 and H3K9me3 ChIP-seq data in untreated and SETDB1-depleted conditions. Consistent with its described roles, SETDB1 depletion resulted in a reduction of H3K9me3 at LTR elements and a modest reduction at minor target regions such as LINE1 elements (Fig. 3B,D; Matsui et al. 2010; Rowe et al. 2010; Protasova et al. 2021). Tracking these changes, ADNP2 binding was reduced at LTRs and, to a lesser extent, at LINEs (Fig. 3B). This reduction was smaller than the effect of the PxVxL mutation, likely due to incomplete loss of H3K9me3 at these elements. Supporting this interpretation, the change in ADNP2 binding strongly correlated with the change in H3K9me3 levels across the entire data set (Supplemental Fig. S5C).

To further quantify the relationship between ChAHP2 and H3K9me3, we analyzed the HP1 decoupling and SETDB1 depletion data using repeat family-wide and consensus mapping approaches. First, we performed a differential binding analysis between ADNP2 and ADNP2^PxVxL^ mutant ChIPs across all transposon families (Fig. 3C). This revealed a significant binding reduction in the PxVxL mutant for many LTRs and LINE1s. Similarly, binding to satellite repeats (GSAT_MM) was reduced, suggesting that binding to nontransposon heterochromatin is also HP1-dependent. Consistent with the peak-based analyses (Supplemental Fig. S4C–E), no transposon class exhibited significantly increased binding (Fig. 3C). A focused look at representative LTR elements and L1MdA confirmed that ADNP2 binding dropped to input levels at H3K9 trimethylated transposons in the HP1-deficient mutant (Fig. 3D; Supplemental Fig. S5D). A reduction was also apparent upon SETDB1 depletion, again smaller in scale. Finally, there was no additional reduction in ADNP2 ChIP signal when SETDB1 was depleted in the ADNP2^PxVxL^ background (Fig. 3D), suggesting that the two perturbations exert their effect via a shared molecular axis. Overall, these data demonstrate that HP1β-mediated binding of H3K9me3 nucleosomes targets ChAHP2 to heterochromatinized retrotransposons and potentially to all other heterochromatic regions.

### ChAHP partially colocalizes with ChAHP2 at retrotransposons

**Figure 4.**
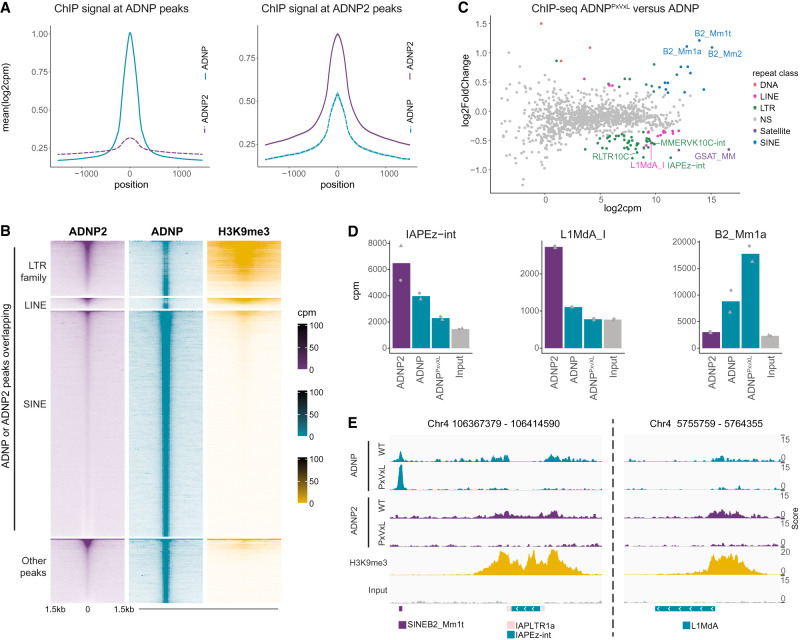
ChAHP and ChAHP2 colocalize at heterochromatin. (*A*) Metaplots of ChIP-seq counts normalized to library size over ADNP peaks or ADNP2 peaks as indicated (mean ± SD; *n* = 2). (*B*) Waterfall plot for ChIPs, centered on a concatenated set of ADNP and ADNP2 peak summits and split by overlap with select repeat annotations as indicated (mean; *n* = 2). (*C*) Differential binding analysis of summed reads over repeat annotations normalized to library size comparing ADNP^PxVxL^ ChIP and WT controls (*n* = 2). (*D*) Summed reads over repeat annotations normalized to library size for input and a series of ChIP samples as indicated (mean; *n* = 2; replicates are represented as points). (*E*) IGV genome browser shots for select regions for ChIPs and input as indicated.

These chromatin binding characteristics appear overall distinct from ChAHP, which has been shown to predominantly bind SINEs (Ostapcuk et al. 2018; Kaaij et al. 2019). We saw little evidence of ADNP2 binding at ADNP-associated SINEs, with few ADNP2 peaks overlapping these transposons and very little ADNP2 signal at ADNP peaks (Figs. 2C,D, 4A,B,E). Conversely, some ADNP does colocalize with ADNP2, specifically at regions marked by H3K9me3, though this binding is less strong than on SINEs (Fig. 4A,B). Indeed, we observed ADNP peaks (Supplemental Table S4) at select heterochromatic repeat regions more frequently than a randomized peak set (Supplemental Fig. S6A). ADNP has been previously reported to bind to H3K9me3-modified chromatin and pericentromeric heterochromatin in an HP1-dependent manner (Mosch et al. 2011; Ostapcuk et al. 2018), prompting us to assess whether ADNP is targeted to ADNP2-bound sites via the same mechanism. To do this, we generated ADNP^PxVxL^ motif point mutants (Supplemental Fig. S6B) and performed ChIP-seq. Abrogating the HP1 interaction in this way resulted in a loss of ADNP signal specifically at H3K9 trimethylated transposons, including LTR and LINE1 families, which are bound by ADNP2 (Fig. 4C,E). Binding to satellite repeats was also significantly reduced, orthogonally validating previously published imaging data (Fig. 4C; Mosch et al. 2011). A focused look at representative heterochromatinized repeats confirmed that ADNP binding at these sites drops to near-background levels after HP1 decoupling (Fig. 4D; Supplemental Fig. S6C). Conversely, binding to SINEs is increased under these conditions (Fig. 4B–D; Supplemental Fig. S6C). This striking observation indicates that ChAHP features two chromatin binding modes: one via HP1 and H3K9me3 to heterochromatin analogous to ChAHP2, and a second H3K9me3-independent mechanism through sequence-specific recruitment to euchromatic SINEs (Mosch et al. 2011; Ostapcuk et al. 2018). Thus, these analyses demonstrate that ChAHP and ChAHP2 have different combinations of chromatin binding specificities, with a notable overlap at heterochromatin.

### Repressive activities of ChAHP and ChAHP2 partially overlap at LINE1 and LTR retrotransposons

The chromatin binding characteristics of the two ChAHP complexes prompted us to assess their individual and combined contributions to transposon silencing. We attempted to generate *adnp/adnp2* double-KO cells but were unable to obtain homozygous clones. To circumvent this issue, we generated cells that allow inducible degradation of ADNP in an *adnp2*^−/−^ background. Approximating a constitutive KO situation, we induced ADNP degradation in *adnp2*^−/−^ cells continuously over 14 days of passaging and confirmed the efficacy of degradation by Western blotting (Supplemental Fig. S7A,B). At the RNA level, consistent with previous data, 36 genes were either upregulated or downregulated upon removal of ADNP (Supplemental Fig. S7C; Supplemental Table S5; Ostapcuk et al. 2018). Genetic KO of *adnp2* caused misregulation of several hundred genes, with a slight tendency for upregulation (Supplemental Fig. S7C; Supplemental Table S5). Depletion of ADNP in *adnp2*^−/−^ cells resulted in misregulation of several hundred genes in addition to those already observed when only ADNP2 was removed, revealing a synthetic effect (Supplemental Fig. S7C; Supplemental Table S5). None of the changing gene categories showed strong and significant patterns in terms of either GO term enrichment (Supplemental Fig. S7D), distance between the promoter and the nearest ADNP/ADNP2 peaks (Supplemental Fig. S7E), or direct association between the peaks and promoters (hypergeometric test, *P* < 0.01). Therefore, ChAHP and ChAHP2 likely do not regulate these genes directly.

**Figure 5.**
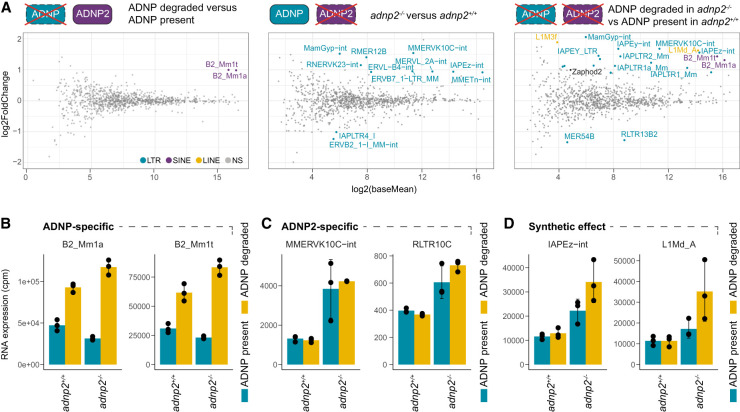
ChAHP and ChAHP2 repress distinct and shared repeat classes. (*A*) Differential expression analysis for repeat families between ADNP/ADNP2 perturbation and the corresponding unperturbed controls (*n* = 3). Significant hits are highlighted with colors (FDR < 0.05; |log_2_FoldChange| > 0.9). ADNP depletion was performed using 250 nM dTAG13 for 14 days whereas ADNP2 was genetically removed using CRISPR–Cas9. (*B–D*) RNA expression normalized to library size for example repeat classes (mean ± SD; *n* = 3) from the same data set as in *A*, representing classes that are specifically ADNP-dependent (*B*), specifically ADNP2-dependent (*C*), or cooperatively regulated (*D*).

We next explored whether any repetitive elements were differentially expressed under the same conditions (Fig. 5A; Supplemental Table S6). Consistent with previous findings, only SINE B2 elements were significantly upregulated upon ADNP depletion (Fig. 5B; Kaaij et al. 2019). Conversely, nine repeat families were significantly upregulated in *adnp2*^−/−^ cells (FDR < 0.05, |log_2_FoldChange > 0.9), while two were downregulated (Fig. 5A). All these differentially expressed repeats belonged to the LTR class of retrotransposons, including families identified as ADNP2-bound in ChIP-seq, such as those corresponding to components of IAP elements and MMERVK10C. Notably, both the internal sequences of the elements and their associated LTR sequences were upregulated, suggesting that all components of these elements increase in expression. We obtained nearly identical findings when intron-associated repeat elements were excluded from the analyses (data not shown), indicating that the observed effects are not covariates of changes in gene expression. Strikingly, combined removal of ADNP and ADNP2 resulted in magnified upregulation of LTR elements when compared with ADNP or ADNP2 removal in isolation. In addition, two LINE1 element subclasses exhibited significant upregulation, with the more prominent being L1MdA. Closer inspection revealed that L1MdA elements are already slightly upregulated upon ADNP2 KO, but the upregulation only reaches significance upon removal of both ADNP and ADNP2 (Fig. 5D). Synergistic regulation was also observed for IAP elements. In contrast, MMERVK10C derepression was exclusively ADNP2-sensitive despite also being weakly bound by ADNP (Fig. 5C). Overall, there was little to no difference in H3K9me3, ADNP, and ADNP2 levels or chromatin accessibility between ERVK insertions that were sensitive to either only ADNP2 or combined ADNP/ADNP2 loss (Supplemental Fig. S8A). Finally, elements that were already upregulated upon ADNP2 loss tended to be more divergent, though this trend was not particularly prominent (Supplemental Fig. S8A).

Derepression of retrotransposons could be caused by compromised H3K9 trimethylation upon loss of ADNP and ADNP2. To assess this, we performed H3K9me3 ChIP-seq under ADNP and ADNP2 perturbation conditions. H3K9me3 levels at ADNP and ADNP2 peaks were not reduced by either individual or combined removal of these factors (Supplemental Fig. S8B,C). We further compared the ADNP/ADNP2-dependent derepression with gross perturbation of H3K9me3 by degrading SETDB1 (Supplemental Fig. S8D). Expression of SINEs was unaffected by loss of SETDB1, whereas LTR elements were upregulated more than an order of magnitude. L1MdA expression was also slightly elevated upon SETDB1 depletion, consistent with previous reports suggesting that SETDB1 plays a small role in H3K9 trimethylation and repression of LINE1 elements (Matsui et al. 2010; Liu et al. 2018). Collectively, these data suggest that ChAHP and ChAHP2 contribute to repression of a variety of retrotransposons with distinct but partially overlapping specificities.

## Discussion

Here we have shown that ADNP2, CHD4, and HP1β coalesce to form ChAHP2, a novel stable protein complex unifying different chromatin regulatory activities. The chromatin binding properties of ChAHP2 are largely distinct from ChAHP in that its predominant recruitment mode is via HP1 and H3K9me3. In contrast to ChAHP, ChAHP2 does not bind euchromatic SINEs but is instead targeted to H3K9me3-modified LTR and LINE1 elements. In line with this sequence-agnostic recruitment mechanism, we were unable to define a sequence motif that would broadly explain the distribution and specificity of the ADNP2 ChIP-seq signal. It is possible that multiple different sequences can be bound by distinct DNA binding domains within ADNP2 (zinc fingers or homeodomain) and contribute weak but important specificity toward certain repetitive elements. Thorough in vitro biochemistry and potentially structural analyses will be required to address this question in the future. Nevertheless, the observed chromatin binding appears consequential, as removal of ADNP2 results in specific upregulation of a subset of ChAHP2-bound retrotransposons.

We also found a supporting role of ChAHP in regulating these elements. Previous studies had reported weak and promiscuous binding of ChAHP to H3K9me3-modified heterochromatin without a clear functional implication (Mosch et al. 2011; Ostapcuk et al. 2018). Since ChAHP binding at non-H3K9me3-modified cognate targets is increased upon disrupting HP1 interaction and thus H3K9me3 binding, a substantial fraction of ChAHP is likely associated with heterochromatin. This observation indirectly supports a role for ChAHP in regulation of heterochromatic loci and clarifies the biological requirement for this chromatin binding modality within the complex. This was previously unappreciated because derepression of heterochromatic targets only becomes apparent when both ADNP and ADNP2 are removed. Notably, the magnitude of this derepression is comparable with the contribution of other factors with proposed roles in LTR regulation such as MORC3, ATRX–DAXX, SMARCAD1, m^6^A methylation, or the recently identified TNRC18 (Sadic et al. 2015; Sachs et al. 2019; Chelmicki et al. 2021; Groh et al. 2021; Liu et al. 2021; Zhao et al. 2023). Thus, we surmise that ChAHP and ChAHP2 constitute one part of a multicomponent retrotransposon repression machinery.

Given that loss of ChAHP/ChAHP2 does not result in compromised H3K9 trimethylation, the most likely mechanism that would confer repressive activities to ChAHP complexes is chromatin remodeling by CHD4. Previously, CHD4 has been shown to contribute to transcriptional silencing via chromatin remodeling activity promoting increased nucleosome densities in a nonpositioned manner at its target loci (Morris et al. 2014; de Dieuleveult et al. 2016; Bornelöv et al. 2018). Such a mechanism would conform to the general concept of transposon control, in which a diverse group of specificity factors guides the activity of less specific chromatin-modifying effectors. This model is typified by the canonical LTR repression system harnessing sequence-specific KRAB-ZFPs to guide the activity of H3K9 methyltransferases. There are two notable differences between this system and ADNP/ADNP2. First, chromatin remodeling activity is stably incorporated into the ChAHP complexes, while the KRAB-ZFPs transiently interact with chromatin modifiers and do not appear to form stable multisubunit complexes (Helleboid et al. 2019). Second, KRAB-ZFPs rapidly evolve and massively multiply to target newly invading TEs (Imbeault et al. 2017; de Tribolet-Hardy et al. 2023), whereas ADNP and ADNP2 are remarkably well conserved since their inception in agnathans. Instead, the recognition of potentially harmful genetic elements in a largely DNA sequence-agnostic manner, which is exerted by the H3K9me3 affinity of HP1 proteins within the ChAHP complexes, is conceptually reminiscent of the HUSH complex (Tchasovnikarova et al. 2015; Seczynska et al. 2022). HUSH recognizes nascent intronless transcripts (a feature typical for TEs) and chromatin modifications rather than the DNA sequence itself. However, like KRAB-ZFPs, HUSH interacts with chromatin modifiers only transiently. Thus, ChAHP and ChAHP2 possess a unique combination of chromatin binding modes and functional properties representing previously unknown components of the LTR, LINE1, and SINE repression machineries.

## Materials and methods

### Cell culture

Mouse embryonic stem cells (129 × C57BL/6 background) with BirA and Cre insertions in the Rosa26 locus (Flemr and Bühler 2015; Ostapcuk et al. 2018) were cultured on gelatin-coated dishes in ES medium containing DMEM (Gibco 21969-035) supplemented with 15% fetal bovine serum (FBS; Gibco), 1× nonessential amino acids (Gibco), 1 mM sodium pyruvate (Gibco), 2 mM L-glutamine (Gibco), 0.1 mM 2-mercaptoethanol (Sigma), 50 mg/mL penicillin, 80 mg/mL streptomycin, 3 μM glycogen synthase kinase (GSK) inhibitor (Calbiochem D00163483 or Sigma CHIR99021), 10 μM MEK inhibitor (Tocris PD0325901), and homemade LIF at 37°C in 5% CO_2_.

### Genome editing

Cells were trypsinized, counted, seeded, and immediately transfected using Lipofectamine 3000 (Invitrogen) according to the manufacturer's instructions. Generally, 300,000 cells were seeded in 6 well plates and transfected with a total of 1–1.5 μg of DNA. For genetic knockout, plasmids encoding gRNAs targeting the N terminus, C terminus, Cas9, and a puromycin resistance cassette were cotransfected for 24 h before selection with 2 mg/mL puromycin for a further 24–36 h. For endogenous tagging, a plasmid harboring a desired homology repair cassette was included. Combined CRISPR–Cas9/TALEN editing was done as described previously (Ostapcuk et al. 2018). The cells were then trypsinized and counted, and 15,000 cells were seeded on a 10 cm dish for colony formation without puromycin selection. When colonies were sufficiently large (4–8 days), they were manually picked and split into two 96 well plates for screening and expansion. Screenings for both positive editing events and unedited loci were performed by PCR and Sanger sequencing of the products. Where applicable, further confirmation was done using Western blotting, RT-qPCR, and RNA sequencing.

### In vitro protein purification and analysis

For cloning, cDNA encoding full-length human ADNP2 (amino acid residues 1–1131) was PCR-amplified and cloned into a pFast-Bac-derived vector (Invitrogen) in-frame with an N-terminal His6 tag. An expression construct encoding full-length human HP1β (amino acid residues 1–185) was generated by amplification of cDNA and cloning into a pFast-Bac-derived vector in-frame with an N-terminal Strep tag II. cDNA encoding for full-length human CHD4 (amino acid residues 1–1912) was amplified and cloned into a pAC8-derived vector in-frame with an N-terminal His6 tag (Abdulrahman et al. 2009). Baculoviruses for protein expression were generated in *Spodoptera frugiperda* Sf9 cells using the Bac-to-Bac method for pFastBac-derived vectors or by cotransfection with viral DNA for pAC8-based vectors. After one round of virus amplification in Sf9 cells, *Trichoplusia ni* High5 cells were infected with the respective baculovirus (150 µL of virus per 10 mL of High5 cells at a density of 2 × 10^6^ cells/mL) and collected 48 h after infection. Cells were lysed by sonication in 50 mM Tris (pH 7.5), 300 mM NaCl, 5 mM β-mercaptoethanol, 0.1% Triton X-100, 1 mM PMSF, and 1× PIC (Sigma-Aldrich). The cleared lysate was passed over a Strep-Tactin sepharose (IBA) column. The bound complex was eluted in 50 mM Tris-HCl (pH 7.5), 100 mM NaCl, 5 mM β-mercaptoethanol, and 2.5 mM desthiobiotin.

### Analytical size exclusion chromatography coupled to multiangle light scattering (SEC-MALS)

Thirty-eight microliters of sample at ∼4–5 mg/mL of protein was injected onto a Superose 6 Increase 10/300 GL column (Cytiva) in 50 mM HEPES-OH (pH 7.4), 150 mM NaCl, and 0.5 mM TCEP using an Agilent Infinity 1260 II HPLC system. In-line refractive index and light-scattering measurements were performed using a Wyatt Optilab T-rEX refractive index detector and a Wyatt miniDAWN Treos 3 light-scattering detector. System control and analysis were carried out using Wyatt Astra 7.3.1 software. System performance was checked with BSA.

### Western blotting

Protein samples in lauryl-dodecyl sulfate (LDS) sample buffer were separated by standard SDS-PAGE on 4%–12% Bis-Tris gradient gels (Novex Bolt, Invitrogen). Separated proteins were transferred onto PVDF membranes (Milipore) in transfer buffer (Bjerrum–Schaeffer–Nielsen + 0.4% SDS) using a semidry transfer procedure (TransBlot Turbo, Bio-Rad) with transfer parameters 1.3 A, 25 V, and 12 min for one gel. The membranes were blocked in 3%–5% skim milk (Sigma) dissolved in Tris-buffered saline + 0.2% (v/v) Tween-20 (TBST). Antibody incubations were performed with antibodies diluted in blocking solution to empirically determined concentrations for a minimum of 1 h up to a maximum of 24 h. When reprobing, membranes were first treated with 0.01% NaN_3_ for 30–60 min to quench the HRP from previous staining rounds. Between each antibody incubation, the membranes were washed for a minimum of 45 min in TBST with at least four buffer exchanges. The horseradish peroxidase system (Immobilon, Milipore) coupled to camera-based detection (Agilent Technologies AI600) was used to visualize protein bands.

### Immunoprecipitations

Cells from one 10 cm dish per condition were harvested by trypsinization before centrifugation at 200*g* for 5 min. Cells were washed once in room temperature PBS and snap-frozen or immediately taken forward for lysis. Pellets were lysed in NP-40 lysis buffer supplemented with protease inhibitors and benzonase (20 mM Tris-HCl at pH 7.4, 150 mM NaCl, 1% [v/v] NP-40, 0.1% [v/v] sodium deoxycholate,1× HALT protease inhibitor cocktail, 200 U of Turbo benzonase [Milipore]) for 30 min at 12°C and cleared by centrifugation at16,000*g* for 20 min at 4°C. Protein concentration in the lysates was determined by Bradford assay against BSA, and 1–3 mg of protein lysate equivalent was loaded onto 10 μL of bead slurry (Dynabeads, GE healthcare) prewashed twice with lysis buffer. For FLAG IPs, 2 μg of antibody was added per 1 mg of protein. Beads were incubated with lysate for 2 h at 4°C and washed twice with lysis buffer and twice with wash buffer (20 mM Tris-HCl at pH 7.4, 150 mM NaCl, 0.1% [v/v] NP-40) before addition of LDS. All bead separation steps were done using magnetic racks.

### Proteomics

For IP-MS, immunoprecipitations were performed as normal with the addition of two washing steps without detergent (20 mM Tris at pH 7.5, 150 mM NaCl), followed by on-bead digestion. IP-MS in Figure 1B was exceptionally performed in 350 mM NaCl, and streptavidin M280 Dynabeads (Invitrogen) were used for pull-down, with all other steps remaining the same. Beads were resuspended by vortexing in 5 µL of digestion buffer (3 M GuaHCl, 20 mM EPPS at pH 8.5, 10 mM CAA, 5 mM TCEP), and 1 µL of 0.2 mg/mL LysC protease (Promega) in 50 mM HEPES (pH 8.5) was added. Proteins were digested for 2 h at room temperature with rotation. The samples were diluted with 17 µL of 50 mM HEPES (pH 8.5) and digested with 1 µL of 0.2 mg/mL trypsin (Promega) in 0.2 mM HCl at 37°C with interval mixing at 2000 rpm for 30 sec every 15 min.

For Figure 1B, digested peptides were acidified with 0.8% TFA (final) and analyzed by LC–MS/MS on an EASY-nLC 1000 (Thermo Scientific) with a two-column setup. Peptides were applied on an Acclaim PepMap 100 (Thermo Scientific) C18 trap column (75 µm ID × 2 cm, 3 µm) in 0.1% formic acid and 2% acetonitrile in H_2_O at a constant pressure of 80 MPa and separated by a linear gradient of 3 min of 2%–6% buffer B in buffer A; 40 min of 6%–22% , 9 min of 22%–28%, 8 min of 28%–36%, and 1 min of 36%–80% buffer B in buffer A; and 14 min of 80% buffer B in buffer A (buffer A: 0.1% formic acid; buffer B: 0.1% formic acid in acetonitrile) on an EASY-Spray column ES801 (50 µm ID × 15 cm, 2 µm) (Thermo Scientific) mounted on a DPV ion source (New Objective) connected to an Orbitrap Fusion (Thermo Scientific) at a flow rate of 150 μL/min. Data were acquired using 120,000 resolution for the peptide measurements in the Orbitrap and a top T (3 sec) method with HCD fragmentation for each precursor and fragment measurement in the ion trap following the manufacturer's guidelines (Thermo Scientific).

For Supplemental Figure S2, we used an Orbitrap Fusion LUMOS (Thermo Fisher Scientific) with VanquishNeo-nLC and an easy source with a 75 μm × 15 cm EasyC18 column. The samples were loaded onto a C18 0.3 mm × 5 mm trap, and backward flush was used for the analysis. The gradients used were 0–3 min in 2%–4% buffer B in buffer A; and 3–43 min in 4%–20%, 43–58 min in 20%–30%, 58–66 min in 30%–36%, 66–68 min in 36%–45%, 68–69 min in 45%–100%, and 69–75 min in 100% buffer A (0.1%FA in H_2_O) and buffer B (0.1%FA, 80% MeCN in H_2_O) at room temperature, and the flow rate during the gradient was 350 μL/min.

Peptides were identified with MaxQuant version 1.5.3.8 using the search engine Andromeda (Cox et al. 2011). The mouse subset of the UniProt version 2017_04 or 2021_05 combined with the contaminant DB from MaxQuant was searched, and the protein and peptide FDR values were set to 0.05. Statistical analysis was done in Perseus version 1.5.2.6 (Tyanova et al. 2016) or using limma within the einProt package (version 0.5.13) (Soneson et al. 2023). Results were filtered to remove reverse hits, contaminants, and peptides found in only one sample. Missing values were imputed, and potential interactors were visualized in volcano plots.

### ChIP-seq

ChIP experiments were performed with at least two different clones from endogenously tagged cell lines. For ADNP and ADNP2, the ChIPs were performed using antibodies against the affinity tag, and for H3K9me3, the ChIPs were performed using a specific antibody as detailed further below. Harvesting was performed by trypsinization, and cells were counted for each sample. For ADNP2 ChIPs, 2 × 10^7^ cells were collected, and for all other ChIPs, 1 × 10^7^ were collected The cells were cross-linked for 8 min at room temperature in 10 mL of PBS supplemented with 1% formaldehyde (Sigma F8775). Cross-linking was quenched by adding glycine to a final concentration of 0.125 mM and incubating for 1 min at room temperature and for 3 min on ice. Cells were pelleted by centrifugation at 500g for 3 min at 4°C, and the pellet was lysed in 10 mL of lysis buffer A (50 mM HEPES at pH 8.0, 140 mM NaCl, 1 mM EDTA, 10% glycerol, 0.5% NP40, 0.25% Triton X-100) for 10 min on ice. After centrifugation, the pellet was resuspended in 10 mL of buffer B (10 mM Tris at pH 8, 1 mM EDTA, 0.5 mM EGTA, 200 mM NaCl) and incubated for 5 min on ice. The samples were centrifuged at 500g for 3 min at 4°C, and the pellets lysed in 180 μL of buffer C (50 mM Tris at pH 8, 5 mM EDTA, 1% SDS, 100 mM NaCl) for 2 min at room temperature and for 10 min on ice. The lysates were diluted in 1.6 mL of ice-cold TE buffer and sonicated in 15 mL tubes twice for 10 cycles of 30 sec on/30 sec off at 4°C (Bioruptor Pico, Bio-Rad). Next, 200 μL of 10× ChIP buffer (0.1% SDS, 10% Triton X-100, 12 mM EDTA, 167 mM Tris-HCl at pH 8, 1.67 M NaCl) was added, and the chromatin was transferred into 2 mL Eppendorf tubes before centrifugation at 16,000 g for 10 min at 4°C. Five percent sheared chromatin was reserved for the input control, while the rest was transferred into fresh tubes. Generally, beads were prewashed in 1× ChIP buffer and added to the sonicated chromatin alongside different amounts of antibody, depending on the ChIP. For ADNP2, we used 20 μL of Protein G Dynabeads and 2 μL of a-FLAG. For H3K9me3, we used 20 μL of Protein G Dynabeads and 2 μL of a-H3K9me3. For ADNP, 20 μL of Protein G Dynabeads and 20 μL of Protein A Dynabeads were premixed and washed twice with 1× ChIP buffer, and 3 μL of a-FLAG was used. Samples were incubated for 4 h at 4°C. ChIPs were washed for 1 min each for each step, four times with RIPA (10 mM Tris-HCl at pH 8.0, 1 mM EDTA at pH 8.0, 140 mM NaCl, 1% Triton X-100, 0.1% SDS, 0.1% Na-deoxycholate), twice with RIPA500 (10 mM Tris-HCl at pH 8.0, 1 mM EDTA at pH 8.0, 500 mM NaCl, 1% Triton X-100, 0.1% SDS, 0.1% Na-deoxycholate), twice with Li wash buffer (10 mM Tris-HCl at pH 8.0, 1 mM EDTA at pH 8.0, 250 mM LiCl, 0.5% NP-40, 0.5% Na-deoxycholate), and once with TEplus (10 mM Tris-HCl at pH 8.0, 1 mM EDTA). Beads were transferred to a fresh tube during the last wash, and wash buffer was completely removed before adding 75 μL of elution buffer (10 mM Tris-HCl at pH 8.0, 1 mM EDTA at pH 8.0, 150 mM NaCl, 1% SDS) and incubating for 20 min at 65°C with constant shaking. Elution was repeated once more with 75 μL of elution buffer for 20 min, eluates were pooled, and 2 μL of 20 μg/μL RNase A was added and incubated for 1 h at 37°C. Next, 2 μL of 20 mg/mL Proteinase K was added, and samples were incubated for 2 h at 55°C followed by decross-linking for 6 h at 65°C. Input samples were adjusted to 150 μL total volume with elution buffer and processed equivalently to ChIP samples. DNA was purified by adding 30 μL of AMPure XP beads, 9 μL of 5 M NaCl, and 190 μL of isopropanol and incubating for 10 min at room temperature after thorough mixing. The beads were collected on a magnetic rack and washed twice with 80% EtOH, and DNA was eluted in 30 μL of 10 mM Tris (pH 8.0) for 5 min at 37°C. Twenty-five microliters of ChIP DNA or 10 ng of input DNA was used to generate libraries using the NEBNext Ultra II library preparation kit for Illumina (NEB). Reactions were scaled down to half; otherwise, processing was according to the manufacturer's manual. Libraries were sequenced 51 bp paired-end reads on a NovaSeq6000 instrument (Illumina), 75 bp paired-end reads on a NextSeq2000 device (Ilumina), or 50 bp single-end reads on a HiSeq2500 device (Ilumina).

### ATAC-seq

ATAC-seq was performed in biological triplicates as previously described (Ostapcuk et al. 2018).

### RNA-seq

RNA was prepared equally as for RT-qPCR. Libraries were prepared using the Ilumina stranded total RNA library preparation, including a ribosomal RNA depletion step, and were sequenced on the Illumina NovaSeq 6000 (51 nt paired-end reads).

### RT-qPCR

RNA was isolated from cells using the Absolutely RNA miniprep kit (Agilent) according to the manufacturer's instructions, including genomic DNA prefiltering and DNase I treatment. The concentration was determined with the RNA broad range reagents on a Qubit 2.0 system according to the manufacturer's instruction. Reverse transcription was performed by adding Primescript II master mix (Takara) to 1× concentration and incubating for 15 min at 37°C, followed by enzyme inactivation for 5 sec at 85°C. qPCR was performed with the SSO advanced Bio-Rad qPCR master mix using an amount of inactivated RT mixture corresponding to 40–200 ng of total RNA (depending on the experiment) with 0.4 μM primers in a CFX96 system (Bio-Rad). The cycling parameters were always 30 sec at 95°C and 40 cycles of 5 sec at 95°C and 15 sec at 60°C, and melt curve of 65°C–95°C.

### Computational methods

#### Read mapping

Reads were mapped to the mouse mm10 genome or a combined mouse/human (mm10/GRCh38) genome (for the samples including spike-ins) using STAR version 2.7.3 (Dobin et al. 2013), allowing up to 10,000 multimapping reads, reporting one multimapper at a random location for ChIP-seq analyses.

The parameters used for ChIP were STAR ‐‐runMode alignReads ‐‐outSAMtype BAM SortedByCoordinate ‐‐readFilesIn R1.fastq.gz R2.fastq.gz ‐‐readFilesCommand zcat ‐‐genomeDir mm10_hg38Spike_refSTAR ‐‐runThreadN 10 ‐‐alignIntronMax 1 ‐‐alignEndsType EndToEnd ‐‐outFilterType Normal ‐‐seedSearchStartLmax 30 ‐‐outFilterMultimapNmax 10000 ‐‐outSAMattributes NH HI NM MD AS nM ‐‐outMultimapperOrder Random ‐‐outSAMmultNmax 1 ‐‐outSAMunmapped Within ‐‐outFileNamePrefix _ ‐‐clip3pAdapterSeq CTGTCTCTTATACACATCT AGATGTGTATAAGAGACAG ‐‐outBAMsortingBinsN 100.

The parameters used for RNA-seq were STAR ‐‐runMode alignReads ‐‐outSAMtype BAM SortedByCoordinate ‐‐readFilesIn R1.fastq.gz R2.fastq.gz ‐‐readFilesCommand zcat –genomeDir tar2_7_3a_GRCm38.primary_assembly_gencodeM23_spliced_sjdb50 ‐‐runThreadN 10 ‐‐outFilterType BySJout ‐‐outFilterMultimapNmax 10000 ‐‐outFilterMismatchNmax 3 ‐‐winAnchorMultimapNmax 20000 ‐‐alignMatesGapMax 350 ‐‐seedSearchStartLmax 30 alignTranscriptsPerReadNmax 30000 ‐‐alignWindowsPerReadNmax 30000 ‐‐alignTranscriptsPerWindowNmax 300 ‐‐seedPerReadNmax 3000 ‐‐seedPerWindowNmax 300 ‐‐seedNoneLociPerWindow 1000 ‐‐outSAMattributes NH HI NM MD AS nM ‐‐outMultimapperOrder Random ‐‐outSAMmultNmax 10000 ‐‐outSAMunmapped Within.

We used RepeatMasker (options: -species “*Mus musculus*”) (http://repeatmasker.org) to annotate repeats and extract consensus repeat sequences. Reads that overlapped repeat annotations were extracted and aligned to repeat consensus sequences using bowtie2 (version: 2.3.5.1) (Langmead and Salzberg 2012) with options: -q -D 20 -R 3 -N 1 -L 20 -i S,1,0.50 ‐‐local -p 20 ‐‐no-unal.

#### Identification and annotation of ADNP2/ADNP binding sites (peak finding)

To identify peaks in ADNP2 or ADNP ChIPs, we pooled all ChIP replicates and used the callpeak function of MACS2 (version 2.2.7.1) (Zhang et al. 2008). Peaks were resized to span 300 bp around the peak summit. To filter out only significantly enriched peaks, we kept only those peaks where the ChIP was enriched >1.2-fold over input in at least three replicates.

We used Gencode version M23 annotation and defined transcription start sites (TSSs) as 300 bp upstream of the annotated TSSs until the TSS. To find overlaps between peaks and TSSs or repeat elements, we used the Genomic Ranges R package (Lawrence et al. 2013). We also generated 100 sets of randomly distributed regions matching our peak sets in number and size and calculated overlaps with TSSs and repeat annotations using these regions. Repeat annotation overlaps were summed up based on the repeat_name column of the RepeatMasker output (generated as described above).

#### ChIP-seq analysis

To determine differences in ADNP2 or ADNP ChIP signals in WT and mutant cells, we used Quasr (Gaidatzis et al. 2015) to count the number of reads in peaks or repeat elements, and edgeR (Robinson et al. 2010) for differential count analysis and normalization to library size (using, if available, the human spike-in total read counts as library size, TMM normalization, and a prior.count of 3). To display ChIP signal intensities (as counts per million [cpm]) in heat maps or metaplots or to calculate cpm in sliding windows across a chromosome, we used the MiniChip R package (https://github.com/fmi-basel/gbuehler-MiniChip). We used MEME-ChIP version 5.5.5 (Machanick and Bailey 2011) to search for motifs in all 6315 ADNP2 peaks using the sequence 500 bp around the peak summit and the option -maxw 25.

#### RNA-seq analysis

To count the number of uniquely mapping reads in genes, we used featureCounts from the Rsubread (Liao et al. 2019) package on Gencode version M24 gene annotation. We used TEtranscripts (Jin et al. 2015) to quantify read counts within transposable elements (TEs) with a TE annotation for mm10 downloaded from https://labshare.cshl.edu/shares/mhammelllab/www-data/TEtranscripts/TE_GTF/mm10_rmsk_TE.gtf.gz and the Gencode version M24 gene annotation with the options ‐‐format BAM ‐‐sortByPos ‐‐stranded reverse ‐‐mode multi. We then calculated differential expression and counts per million using DESeq2 (Love et al. 2014).

To find enriched gene ontology terms among regulated genes, we used the enrichGO function of the clusterProfiler (Yu et al. 2012) R package.

### Data and code availability

All NGS data generated for this study have been deposited at NCBI GEO and are available under accession number GSE253069. Custom scripts for data analysis are available on GitHub (https://github.com/xxxmichixxx/ChAHP2). The mass spectrometry proteomics data have been deposited to the ProteomeXchange Consortium via the Proteomics Identification Database (Perez-Riverol et al. 2021) partner repository with the data set identifiers PXD048314 and PXD048310. Other tools used are indicated in the respective Materials and Methods sections.

## Supplementary Material

Supplement 1

Supplement 2

Supplement 3

Supplement 4

Supplement 5

Supplement 6

Supplement 7

Supplement 8

Supplement 9

Supplement 10

Supplement 11

Supplement 12

Supplement 13

Supplement 14

Supplement 15

## References

[GAD351769AHEC1] Abdulrahman W, Uhring M, Kolb-Cheynel I, Garnier J-M, Moras D, Rochel N, Busso D, Poterszman A. 2009. A set of baculovirus transfer vectors for screening of affinity tags and parallel expression strategies. Anal Biochem 385: 383–385. 10.1016/j.ab.2008.10.044 19061853

[GAD351769AHEC2] Altenhoff AM, Levy J, Zarowiecki M, Tomiczek B, Vesztrocy AW, Dalquen DA, Müller S, Telford MJ, Glover NM, Dylus D, 2019. OMA standalone: orthology inference among public and custom genomes and transcriptomes. Genome Res 29: 1152–1163. 10.1101/gr.243212.118 31235654 PMC6633268

[GAD351769AHEC3] Aneichyk T, Hendriks WT, Yadav R, Shin D, Gao D, Vaine CA, Collins RL, Domingo A, Currall B, Stortchevoi A, 2018. Dissecting the causal mechanism of X-linked dystonia-parkinsonism by integrating genome and transcriptome assembly. Cell 172: 897–909.e21. 10.1016/j.cell.2018.02.011 29474918 PMC5831509

[GAD351769AHEC4] Ayyanathan K, Lechner MS, Bell P, Maul GG, Schultz DC, Yamada Y, Tanaka K, Torigoe K, Rauscher FJ. 2003. Regulated recruitment of HP1 to a euchromatic gene induces mitotically heritable, epigenetic gene silencing: a mammalian cell culture model of gene variegation. Genes Dev 17: 1855–1869. 10.1101/gad.1102803 12869583 PMC196232

[GAD351769AHEC5] Bannister AJ, Zegerman P, Partridge JF, Miska EA, Thomas JO, Allshire RC, Kouzarides T. 2001. Selective recognition of methylated lysine 9 on histone H3 by the HP1 chromo domain. Nature 410: 120–124. 10.1038/35065138 11242054

[GAD351769AHEC6] Bornelöv S, Reynolds N, Xenophontos M, Gharbi S, Johnstone E, Floyd R, Ralser M, Signolet J, Loos R, Dietmann S, 2018. The nucleosome remodeling and deacetylation complex modulates chromatin structure at sites of active transcription to fine-tune gene expression. Mol Cell 71: 56–72.e4. 10.1016/j.molcel.2018.06.003 30008319 PMC6039721

[GAD351769AHEC7] Castro-Diaz N, Ecco G, Coluccio A, Kapopoulou A, Yazdanpanah B, Friedli M, Duc J, Jang SM, Turelli P, Trono D. 2014. Evolutionally dynamic L1 regulation in embryonic stem cells. Genes Dev 28: 1397–1409. 10.1101/gad.241661.114 24939876 PMC4083085

[GAD351769AHEC8] Chelmicki T, Roger E, Teissandier A, Dura M, Bonneville L, Rucli S, Dossin F, Fouassier C, Lameiras S, Bourc'his D. 2021. M6a RNA methylation regulates the fate of endogenous retroviruses. Nature 591: 312–316. 10.1038/s41586-020-03135-1 33442060

[GAD351769AHEC9] Cox J, Neuhauser N, Michalski A, Scheltema RA, Olsen JV, Mann M. 2011. Andromeda: a peptide search engine integrated into the MaxQuant environment. J Proteome Res 10: 1794–1805. 10.1021/pr101065j 21254760

[GAD351769AHEC10] Daniels GR, Deininger PL. 1985. Repeat sequence families derived from mammalian tRNA genes. Nature 317: 819–822. 10.1038/317819a0 3851163

[GAD351769AHEC11] de Dieuleveult M, Yen K, Hmitou I, Depaux A, Boussouar F, Dargham DB, Jounier S, Humbertclaude H, Ribierre F, Baulard C, 2016. Genome-wide nucleosome specificity and function of chromatin remodellers in ES cells. Nature 530: 113–116. 10.1038/nature16505 26814966 PMC4871117

[GAD351769AHEC12] de Tribolet-Hardy J, Thorball CW, Forey R, Planet E, Duc J, Coudray A, Khubieh B, Offner S, Pulver C, Fellay J, 2023. Genetic features and genomic targets of human KRAB–zinc finger proteins. Genome Res 33: 1409–1423. 10.1101/gr.277722.123 37730438 PMC10547255

[GAD351769AHEC13] Dewannieux M, Heidmann T. 2005. L1-mediated retrotransposition of Murine B1 and B2 SINEs recapitulated in cultured cells. J Mol Biol 349: 241–247. 10.1016/j.jmb.2005.03.068 15890192

[GAD351769AHEC14] Dewannieux M, Esnault C, Heidmann T. 2003. LINE-mediated retrotransposition of marked Alu sequences. Nat Genet 35: 41–48. 10.1038/ng1223 12897783

[GAD351769AHEC15] Dobin A, Davis CA, Schlesinger F, Drenkow J, Zaleski C, Jha S, Batut P, Chaisson M, Gingeras TR. 2013. STAR: ultrafast universal RNA-seq aligner. Bioinformatics 29: 15–21. 10.1093/bioinformatics/bts635 23104886 PMC3530905

[GAD351769AHEC16] Ecco G, Imbeault M, Trono D. 2017. KRAB zinc finger proteins. Development 144: 2719–2729. 10.1242/dev.132605 28765213 PMC7117961

[GAD351769AHEC17] Flemr M, Bühler M. 2015. Single-step generation of conditional knockout mouse embryonic stem cells. Cell Rep 12: 709–716. 10.1016/j.celrep.2015.06.051 26190102

[GAD351769AHEC18] Gaidatzis D, Lerch A, Hahne F, Stadler MB. 2015. Quasr: quantification and annotation of short reads in R. Bioinformatics 31: 1130–1132. 10.1093/bioinformatics/btu781 25417205 PMC4382904

[GAD351769AHEC19] Gonçalves A, Oliveira J, Coelho T, Taipa R, Melo-Pires M, Sousa M, Santos R. 2017. Exonization of an intronic LINE-1 element causing becker muscular dystrophy as a novel mutational mechanism in dystrophin gene. Genes 8: 253. 10.3390/genes8100253 28972564 PMC5664103

[GAD351769AHEC20] Groh S, Milton AV, Marinelli LK, Sickinger CV, Russo A, Bollig H, de Almeida GP, Schmidt A, Forné I, Imhof A, 2021. Morc3 silences endogenous retroviruses by enabling Daxx-mediated histone H3.3 incorporation. Nat Commun 12: 5996. 10.1038/s41467-021-26288-7 34650047 PMC8516933

[GAD351769AHEC21] Hancks DC, Kazazian HH. 2016. Roles for retrotransposon insertions in human disease. Mob DNA 7: 9. 10.1186/s13100-016-0065-9 27158268 PMC4859970

[GAD351769AHEC22] Helleboid P, Heusel M, Duc J, Piot C, Thorball CW, Coluccio A, Pontis J, Imbeault M, Turelli P, Aebersold R, 2019. The interactome of KRAB zinc finger proteins reveals the evolutionary history of their functional diversification. EMBO J 38: e101220. 10.15252/embj.2018101220 31403225 PMC6745500

[GAD351769AHEC23] Helsmoortel C, Vulto-van Silfhout AT, Coe BP, Vandeweyer G, Rooms L, van den Ende J, Schuurs-Hoeijmakers JHM, Marcelis CL, Willemsen MH, Vissers LELM, 2014. A SWI/SNF-related autism syndrome caused by de novo mutations in ADNP. Nat Genet 46: 380–384. 10.1038/ng.2899 24531329 PMC3990853

[GAD351769AHEC24] Imbeault M, Helleboid P-Y, Trono D. 2017. KRAB zinc-finger proteins contribute to the evolution of gene regulatory networks. Nature 543: 550–554. 10.1038/nature21683 28273063

[GAD351769AHEC25] Jin Y, Tam OH, Paniagua E, Hammell M. 2015. TEtranscripts: a package for including transposable elements in differential expression analysis of RNA-seq datasets. Bioinformatics 31: 3593–3599. 10.1093/bioinformatics/btv422 26206304 PMC4757950

[GAD351769AHEC26] Kaaij LJT, Mohn F, van der Weide RH, de Wit E, Bühler M. 2019. The ChAHP complex counteracts chromatin looping at CTCF sites that emerged from SINE expansions in mouse. Cell 178: 1437–1451.e14. 10.1016/j.cell.2019.08.007 31491387

[GAD351769AHEC27] Karimi MM, Goyal P, Maksakova IA, Bilenky M, Leung D, Tang JX, Shinkai Y, Mager DL, Jones S, Hirst M, 2011. DNA methylation and SETDB1/H3K9me3 regulate predominantly distinct sets of genes, retroelements, and chimeric transcripts in mESCs. Cell Stem Cell 8: 676–687. 10.1016/j.stem.2011.04.004 21624812 PMC3857791

[GAD351769AHEC28] Kazazian HH Jr. 2004. Mobile elements: drivers of genome evolution. Science 303: 1626–1632. 10.1126/science.1089670 15016989

[GAD351769AHEC29] Lachner M, O'Carroll D, Rea S, Mechtler K, Jenuwein T. 2001. Methylation of histone H3 lysine 9 creates a binding site for HP1 proteins. Nature 410: 116–120. 10.1038/35065132 11242053

[GAD351769AHEC30] Langmead B, Salzberg SL. 2012. Fast gapped-read alignment with Bowtie 2. Nat Methods 9: 357–359. 10.1038/nmeth.1923 22388286 PMC3322381

[GAD351769AHEC31] Lawrence M, Huber W, Pagès H, Aboyoun P, Carlson M, Gentleman R, Morgan MT, Carey VJ. 2013. Software for computing and annotating genomic ranges. PLoS Comput Biol 9: e1003118. 10.1371/journal.pcbi.1003118 23950696 PMC3738458

[GAD351769AHEC32] Lechner MS, Begg GE, Speicher DW, Rauscher FJ. 2000. Molecular determinants for targeting heterochromatin protein 1-mediated gene silencing: direct chromoshadow domain–KAP-1 corepressor interaction is essential. Mol Cell Biol 20: 6449–6465. 10.1128/MCB.20.17.6449-6465.2000 10938122 PMC86120

[GAD351769AHEC33] Liao Y, Smyth GK, Shi W. 2019. The R package Rsubread is easier, faster, cheaper and better for alignment and quantification of RNA sequencing reads. Nucleic Acids Res 47: gkz114. 10.1093/nar/gkz114 PMC6486549 30783653

[GAD351769AHEC34] Liu N, Lee CH, Swigut T, Grow E, Gu B, Bassik MC, Wysocka J. 2018. Selective silencing of euchromatic L1s revealed by genome-wide screens for L1 regulators. Nature 553: 228–232. 10.1038/nature25179 29211708 PMC5774979

[GAD351769AHEC35] Liu J, Gao M, He J, Wu K, Lin S, Jin L, Chen Y, Liu H, Shi J, Wang X, 2021. The RNA m6A reader YTHDC1 silences retrotransposons and guards ES cell identity. Nature 591: 322–326. 10.1038/s41586-021-03313-9 33658714

[GAD351769AHEC36] Love MI, Huber W, Anders S. 2014. Moderated estimation of fold change and dispersion for RNA-seq data with DESeq2. Genome Biol 15: 550. 10.1186/s13059-014-0550-8 25516281 PMC4302049

[GAD351769AHEC37] Macfarlan TS, Gifford WD, Agarwal S, Driscoll S, Lettieri K, Wang J, Andrews SE, Franco L, Rosenfeld MG, Ren B, 2011. Endogenous retroviruses and neighboring genes are coordinately repressed by LSD1/KDM1A. Genes Dev 25: 594–607. 10.1101/gad.2008511 21357675 PMC3059833

[GAD351769AHEC38] Machanick P, Bailey TL. 2011. MEME-ChIP: motif analysis of large DNA datasets. Bioinformatics 27: 1696–1697. 10.1093/bioinformatics/btr189 21486936 PMC3106185

[GAD351769AHEC39] Madeira F, Pearce M, Tivey ARN, Basutkar P, Lee J, Edbali O, Madhusoodanan N, Kolesnikov A, Lopez R. 2022. Search and sequence analysis tools services from EMBL-EBI in 2022. Nucleic Acids Res 50: W276–W279. 10.1093/nar/gkac240 35412617 PMC9252731

[GAD351769AHEC40] Mandel S, Rechavi G, Gozes I. 2007. Activity-dependent neuroprotective protein (ADNP) differentially interacts with chromatin to regulate genes essential for embryogenesis. Dev Biol 303: 814–824. 10.1016/j.ydbio.2006.11.039 17222401

[GAD351769AHEC41] Matsui T, Leung D, Miyashita H, Maksakova IA, Miyachi H, Kimura H, Tachibana M, Lorincz MC, Shinkai Y. 2010. Proviral silencing in embryonic stem cells requires the histone methyltransferase ESET. Nature 464: 927–931. 10.1038/nature08858 20164836

[GAD351769AHEC42] Mellacheruvu D, Wright Z, Couzens AL, Lambert J-P, St-Denis NA, Li T, Miteva YV, Hauri S, Sardiu ME, Low TY, 2013. The CRAPome: a contaminant repository for affinity purification–mass spectrometry data. Nat Methods 10: 730–736. 10.1038/nmeth.2557 23921808 PMC3773500

[GAD351769AHEC43] Morris SA, Baek S, Sung M-H, John S, Wiench M, Johnson TA, Schiltz RL, Hager GL. 2014. Overlapping chromatin-remodeling systems collaborate genome wide at dynamic chromatin transitions. Nat Struct Mol Biol 21: 73–81. 10.1038/nsmb.2718 24317492 PMC3947387

[GAD351769AHEC44] Mosch K, Franz H, Soeroes S, Singh PB, Fischle W. 2011. HP1 recruits activity-dependent neuroprotective protein to H3K9me3 marked pericentromeric heterochromatin for silencing of major satellite repeats. PLoS ONE 6: e15894. 10.1371/journal.pone.0015894 21267468 PMC3022755

[GAD351769AHEC45] Müller I, Moroni AS, Shlyueva D, Sahadevan S, Schoof EM, Radzisheuskaya A, Højfeldt JW, Tatar T, Koche RP, Huang C, 2021. MPP8 is essential for sustaining self-renewal of ground-state pluripotent stem cells. Nat Commun 12: 3034. 10.1038/s41467-021-23308-4 34031396 PMC8144423

[GAD351769AHEC46] Nabet B, Roberts JM, Buckley DL, Paulk J, Dastjerdi S, Yang A, Leggett AL, Erb MA, Lawlor MA, Souza A, 2018. The dTAG system for immediate and target-specific protein degradation. Nat Chem Biol 14: 431–441. 10.1038/s41589-018-0021-8 29581585 PMC6295913

[GAD351769AHEC47] Ostapcuk V, Mohn F, Carl SH, Basters A, Hess D, Iesmantavicius V, Lampersberger L, Flemr M, Pandey A, Thomä NH, 2018. Activity-dependent neuroprotective protein recruits HP1 and CHD4 to control lineage-specifying genes. Nature 557: 739–743. 10.1038/s41586-018-0153-8 29795351

[GAD351769AHEC48] Perez-Riverol Y, Bai J, Bandla C, García-Seisdedos D, Hewapathirana S, Kamatchinathan S, Kundu DJ, Prakash A, Frericks-Zipper A, Eisenacher M, 2021. The PRIDE database resources in 2022: a hub for mass spectrometry-based proteomics evidences. Nucleic Acids Res 50: D543–D552. 10.1093/nar/gkab1038 PMC8728295 34723319

[GAD351769AHEC49] Platt RN, Vandewege MW, Ray DA. 2018. Mammalian transposable elements and their impacts on genome evolution. Chromosome Res 26: 25–43. 10.1007/s10577-017-9570-z 29392473 PMC5857283

[GAD351769AHEC50] Protasova MS, Andreeva TV, Rogaev EI. 2021. Factors regulating the activity of LINE1 retrotransposons. Genes 12: 1562. 10.3390/genes12101562 34680956 PMC8535693

[GAD351769AHEC51] Qian Y, Mancini-DiNardo D, Judkins T, Cox HC, Brown K, Elias M, Singh N, Daniels C, Holladay J, Coffee B, 2017. Identification of pathogenic retrotransposon insertions in cancer predisposition genes. Cancer Genet 216-217: 159–169. 10.1016/j.cancergen.2017.08.002 29025590

[GAD351769AHEC52] Quenneville S, Turelli P, Bojkowska K, Raclot C, Offner S, Kapopoulou A, Trono D. 2012. The KRAB–ZFP/KAP1 system contributes to the early embryonic establishment of site-specific DNA methylation patterns maintained during development. Cell Rep 2: 766–773. 10.1016/j.celrep.2012.08.043 23041315 PMC3677399

[GAD351769AHEC53] Raiz J, Damert A, Chira S, Held U, Klawitter S, Hamdorf M, Löwer J, Strätling WH, Löwer R, Schumann GG. 2012. The non-autonomous retrotransposon SVA is trans -mobilized by the human LINE-1 protein machinery. Nucleic Acids Res 40: 1666–1683. 10.1093/nar/gkr863 22053090 PMC3287187

[GAD351769AHEC54] Robbez-Masson L, Tie CHC, Conde L, Tunbak H, Husovsky C, Tchasovnikarova IA, Timms RT, Herrero J, Lehner PJ, Rowe HM. 2018. The HUSH complex cooperates with TRIM28 to repress young retrotransposons and new genes. Genome Res 28: 836–845. 10.1101/gr.228171.117 29728366 PMC5991525

[GAD351769AHEC55] Robinson MD, McCarthy DJ, Smyth GK. 2010. edgeR: a Bioconductor package for differential expression analysis of digital gene expression data. Bioinformatics 26: 139–140. 10.1093/bioinformatics/btp616 19910308 PMC2796818

[GAD351769AHEC56] Rowe HM, Jakobsson J, Mesnard D, Rougemont J, Reynard S, Aktas T, Maillard PV, Layard-Liesching H, Verp S, Marquis J, 2010. KAP1 controls endogenous retroviruses in embryonic stem cells. Nature 463: 237–240. 10.1038/nature08674 20075919

[GAD351769AHEC57] Sachs P, Ding D, Bergmaier P, Lamp B, Schlagheck C, Finkernagel F, Nist A, Stiewe T, Mermoud JE. 2019. SMARCAD1 ATPase activity is required to silence endogenous retroviruses in embryonic stem cells. Nat Commun 10: 1335. 10.1038/s41467-019-09078-0 30902974 PMC6430823

[GAD351769AHEC58] Sadic D, Schmidt K, Groh S, Kondofersky I, Ellwart J, Fuchs C, Theis FJ, Schotta G. 2015. Atrx promotes heterochromatin formation at retrotransposons. EMBO Rep 16: 836–850. 10.15252/embr.201439937 26012739 PMC4515123

[GAD351769AHEC59] Schultz DC, Friedman JR, Rauscher FJ. 2001. Targeting histone deacetylase complexes via KRAB–zinc finger proteins: the PHD and bromodomains of KAP-1 form a cooperative unit that recruits a novel isoform of the Mi-2α subunit of NuRD. Genes Dev 15: 428–443. 10.1101/gad.869501 11230151 PMC312636

[GAD351769AHEC60] Schultz DC, Ayyanathan K, Negorev D, Maul GG, Rauscher FJ. 2002. SETDB1: a novel KAP-1-associated histone H3, lysine 9-specific methyltransferase that contributes to HP1-mediated silencing of euchromatic genes by KRAB zinc-finger proteins. Genes Dev 16: 919–932. 10.1101/gad.973302 11959841 PMC152359

[GAD351769AHEC61] Seczynska M, Bloor S, Cuesta SM, Lehner PJ. 2022. Genome surveillance by HUSH-mediated silencing of intronless mobile elements. Nature 601: 440–445. 10.1038/s41586-021-04228-1 34794168 PMC8770142

[GAD351769AHEC62] Smallwood A, Estève P-O, Pradhan S, Carey M. 2007. Functional cooperation between HP1 and DNMT1 mediates gene silencing. Genes Dev 21: 1169–1178. 10.1101/gad.1536807 17470536 PMC1865489

[GAD351769AHEC63] Soneson C, Iesmantavicius V, Hess D, Stadler MB, Seebacher J. 2023. einprot: flexible, easy-to-use, reproducible workflows for statistical analysis of quantitative proteomics data. J Open Source Softw 8: 5750. 10.21105/joss.05750

[GAD351769AHEC64] Tchasovnikarova IA, Timms RT, Matheson NJ, Wals K, Antrobus R, Göttgens B, Dougan G, Dawson MA, Lehner PJ. 2015. Epigenetic silencing by the HUSH complex mediates position-effect variegation in human cells. Science 348: 1481–1485. 10.1126/science.aaa7227 26022416 PMC4487827

[GAD351769AHEC65] Teugels E, De Brakeleer S, Goelen G, Lissens W, Sermijn E, Grève JD. 2005. De novo Alu element insertions targeted to a sequence common to the BRCA1 and BRCA2 genes. Hum Mutat 26: 284–284. 10.1002/humu.9366 16088935

[GAD351769AHEC66] Thiru A, Nietlispach D, Mott HR, Okuwaki M, Lyon D, Nielsen PR, Hirshberg M, Verreault A, Murzina NV, Laue ED. 2004. Structural basis of HP1/PXVXL motif peptide interactions and HP1 localisation to heterochromatin. EMBO J 23: 489–499. 10.1038/sj.emboj.7600088 14765118 PMC1271814

[GAD351769AHEC67] Turelli P, Castro-Diaz N, Marzetta F, Kapopoulou A, Raclot C, Duc J, Tieng V, Quenneville S, Trono D. 2014. Interplay of TRIM28 and DNA methylation in controlling human endogenous retroelements. Genome Res 24: 1260–1270. 10.1101/gr.172833.114 24879559 PMC4120080

[GAD351769AHEC68] Tyanova S, Temu T, Sinitcyn P, Carlson A, Hein MY, Geiger T, Mann M, Cox J. 2016. The Perseus computational platform for comprehensive analysis of (prote)omics data. Nat Methods 13: 731–740. 10.1038/nmeth.3901 27348712

[GAD351769AHEC69] Ullu E, Tschudi C. 1984. Alu sequences are processed 7SL RNA genes. Nature 312: 171–172. 10.1038/312171a0 6209580

[GAD351769AHEC70] Varshney D, Vavrova-Anderson J, Oler AJ, Cowling VH, Cairns BR, White RJ. 2015. SINE transcription by RNA polymerase III is suppressed by histone methylation but not by DNA methylation. Nat Commun 6: 6569. 10.1038/ncomms7569 25798578 PMC4382998

[GAD351769AHEC71] Wang Z, Fan R, Russo A, Cernilogar FM, Nuber A, Schirge S, Shcherbakova I, Dzhilyanova I, Ugur E, Anton T, 2022. Dominant role of DNA methylation over H3K9me3 for IAP silencing in endoderm. Nat Commun 13: 5447. 10.1038/s41467-022-32978-7 36123357 PMC9485127

[GAD351769AHEC72] Waterhouse AM, Procter JB, Martin DMA, Clamp M, Barton GJ. 2009. Jalview version 2—a multiple sequence alignment editor and analysis workbench. Bioinformatics 25: 1189–1191. 10.1093/bioinformatics/btp033 19151095 PMC2672624

[GAD351769AHEC73] Wicker T, Sabot F, Hua-Van A, Bennetzen JL, Capy P, Chalhoub B, Flavell A, Leroy P, Morgante M, Panaud O, 2007. A unified classification system for eukaryotic transposable elements. Nat Rev Genet 8: 973–982. 10.1038/nrg2165 17984973

[GAD351769AHEC74] Wolf D, Goff SP. 2009. Embryonic stem cells use ZFP809 to silence retroviral DNAs. Nature 458: 1201–1204. 10.1038/nature07844 19270682 PMC2676211

[GAD351769AHEC75] Yu G, Wang L-G, Han Y, He Q-Y. 2012. Clusterprofiler: an R package for comparing biological themes among gene clusters. OMICS: A J Integr Biol 16: 284–287. 10.1089/omi.2011.0118 PMC3339379 22455463

[GAD351769AHEC76] Zhang Y, Liu T, Meyer CA, Eeckhoute J, Johnson DS, Bernstein BE, Nusbaum C, Myers RM, Brown M, Li W, 2008. Model-based analysis of ChIP-seq (MACS). Genome Biol 9: R137. 10.1186/gb-2008-9-9-r137 18798982 PMC2592715

[GAD351769AHEC77] Zhao S, Lu J, Pan B, Fan H, Byrum SD, Xu C, Kim A, Guo Y, Kanchi KL, Gong W, 2023. TNRC18 engages H3K9me3 to mediate silencing of endogenous retrotransposons. Nature 623: 633–1642. 10.1038/s41586-023-06688-z 37938770 PMC11000523

