# Supplementary figures and images for: ChAHP2 and ChAHP control diverse retrotransposons by complementary activities
Source: Genes Dev. 2024 Jun 1;38(11-12):554–68. doi: 10.1101/gad.351769.124 (PMC11293393; doi:10.1101/gad.351769.124)

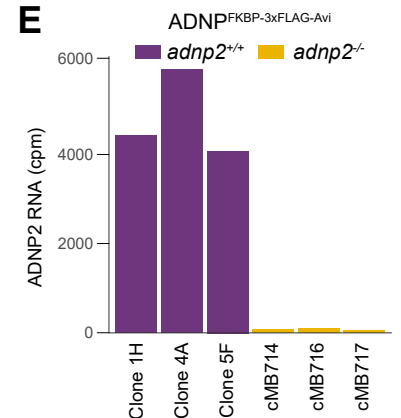

Figure S1

Supplement: Supplement 1 [file Supplemental_FigS1.pdf]

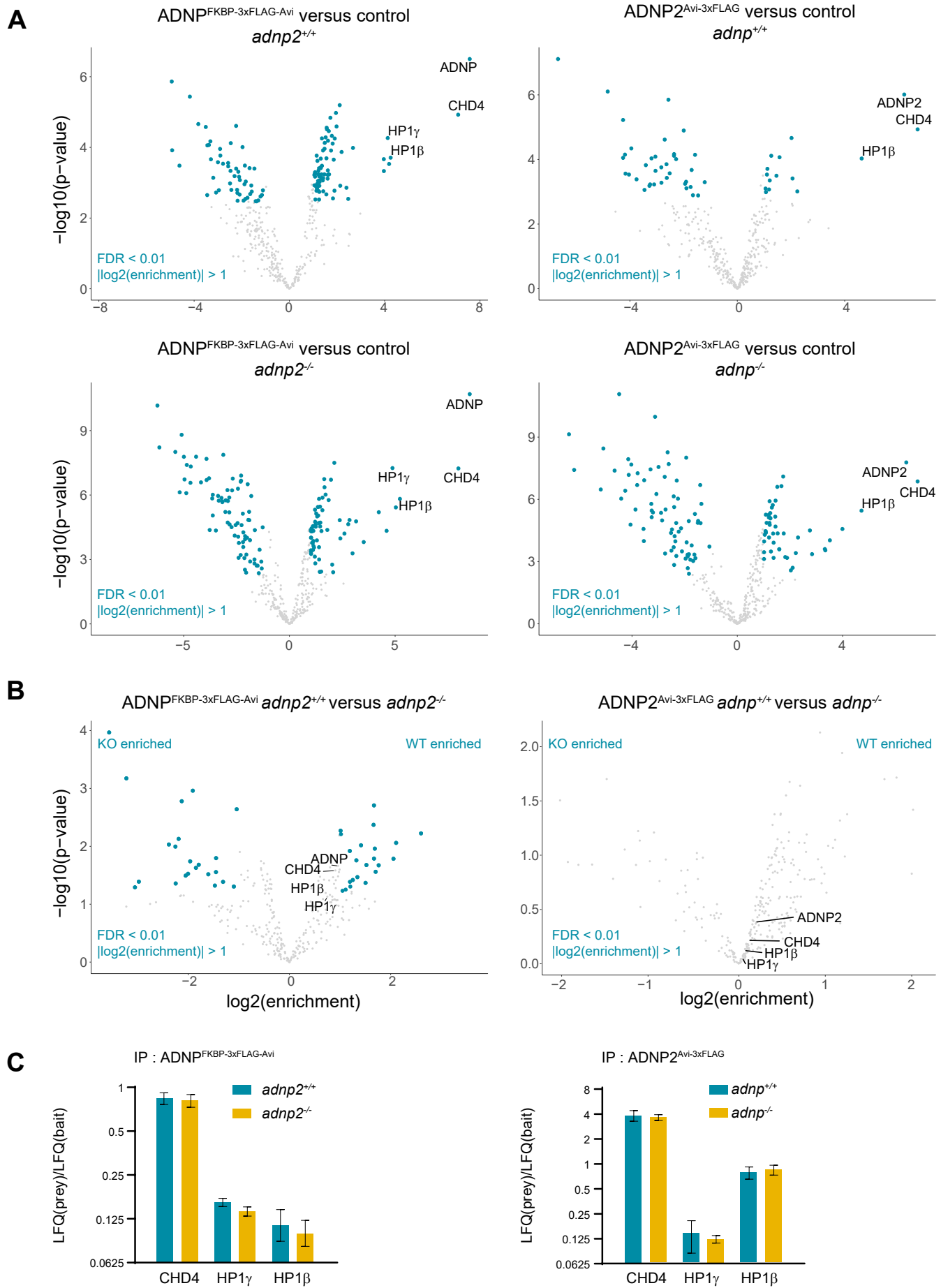

Figure S2

Supplement: Supplement 2 [file Supplemental_FigS2.pdf]

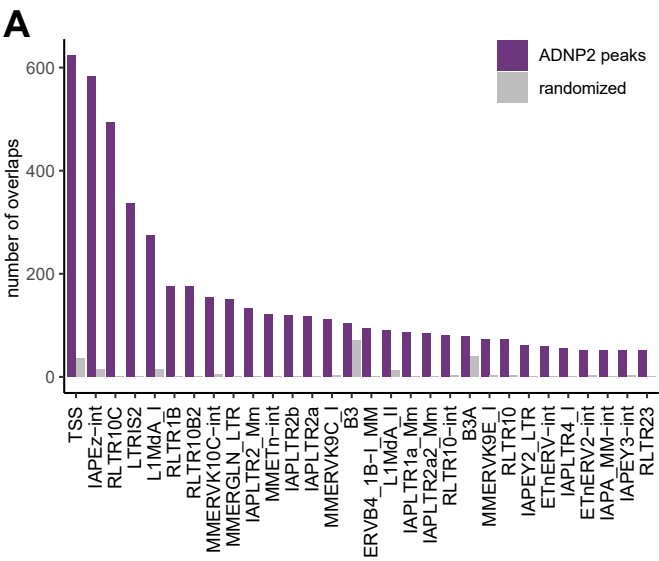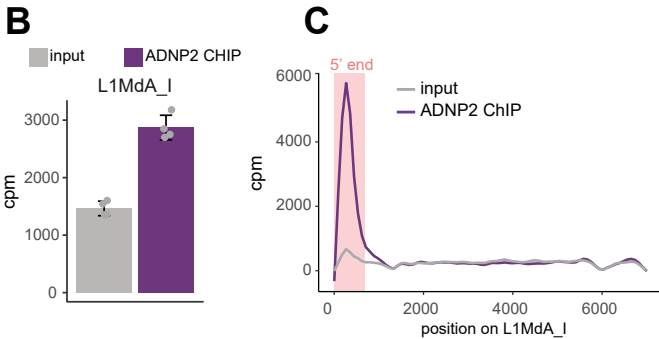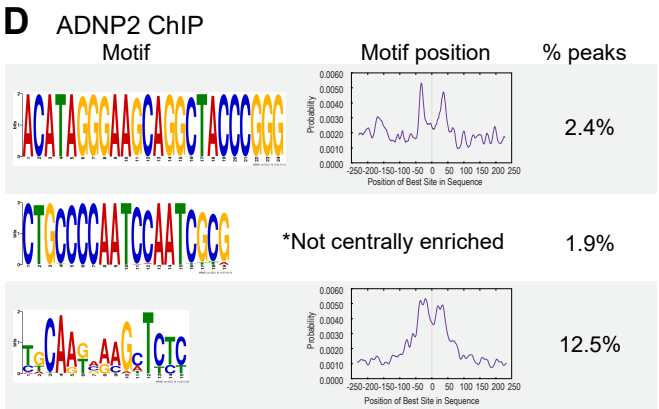

Supplement: Supplement 3 [file Supplemental_FigS3.pdf]

**A**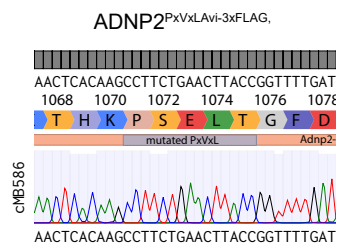**B**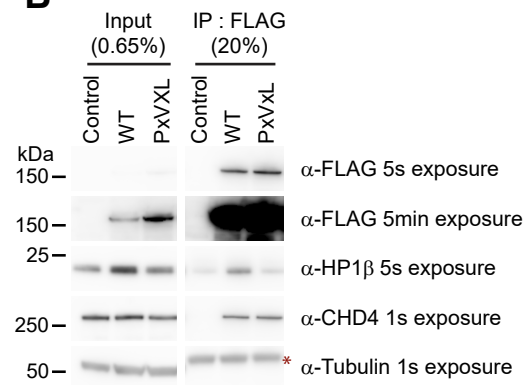**C**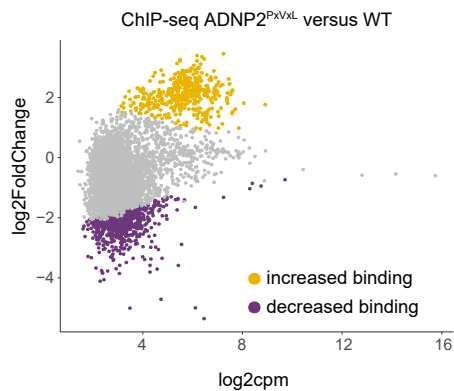**D**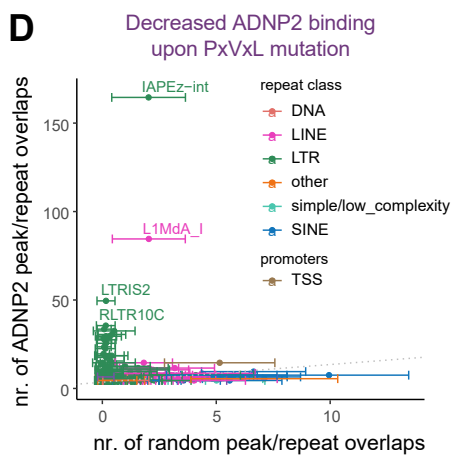**E**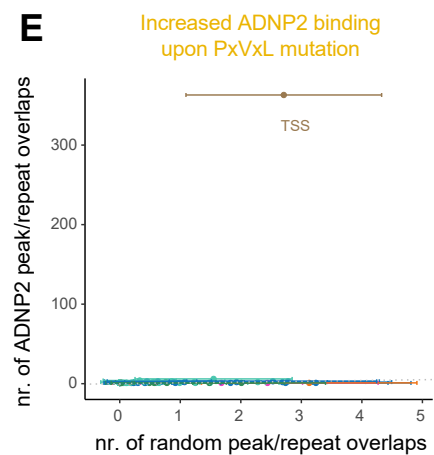**F**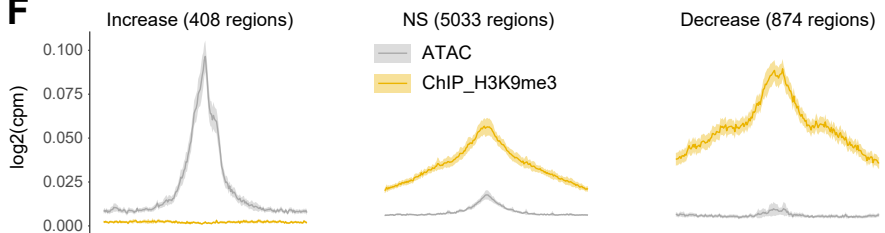**G**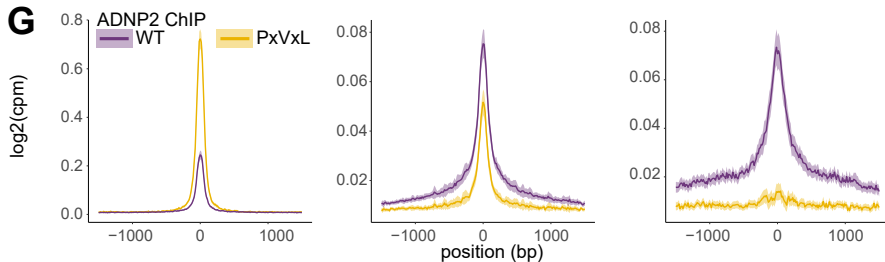

Supplement: Supplement 4 [file Supplemental_FigS4.pdf]

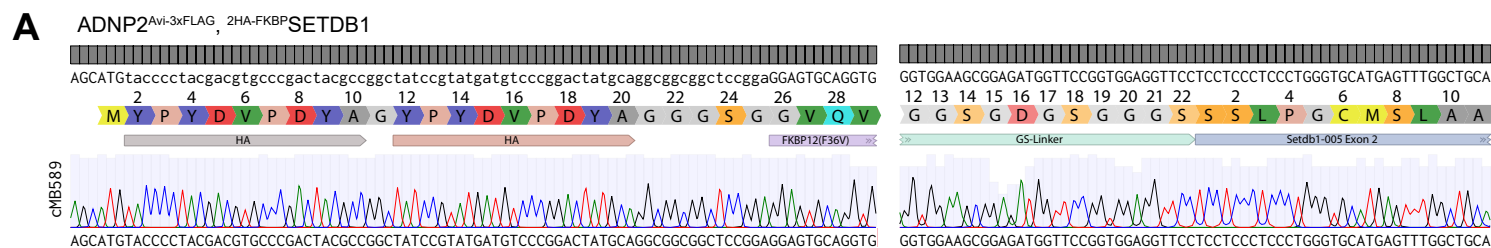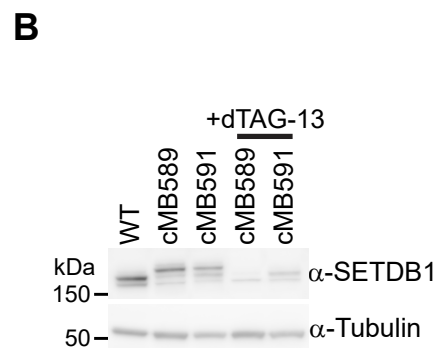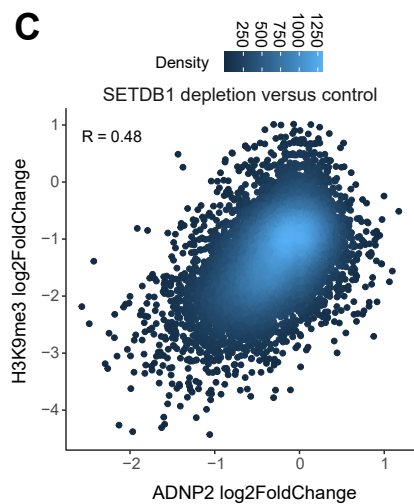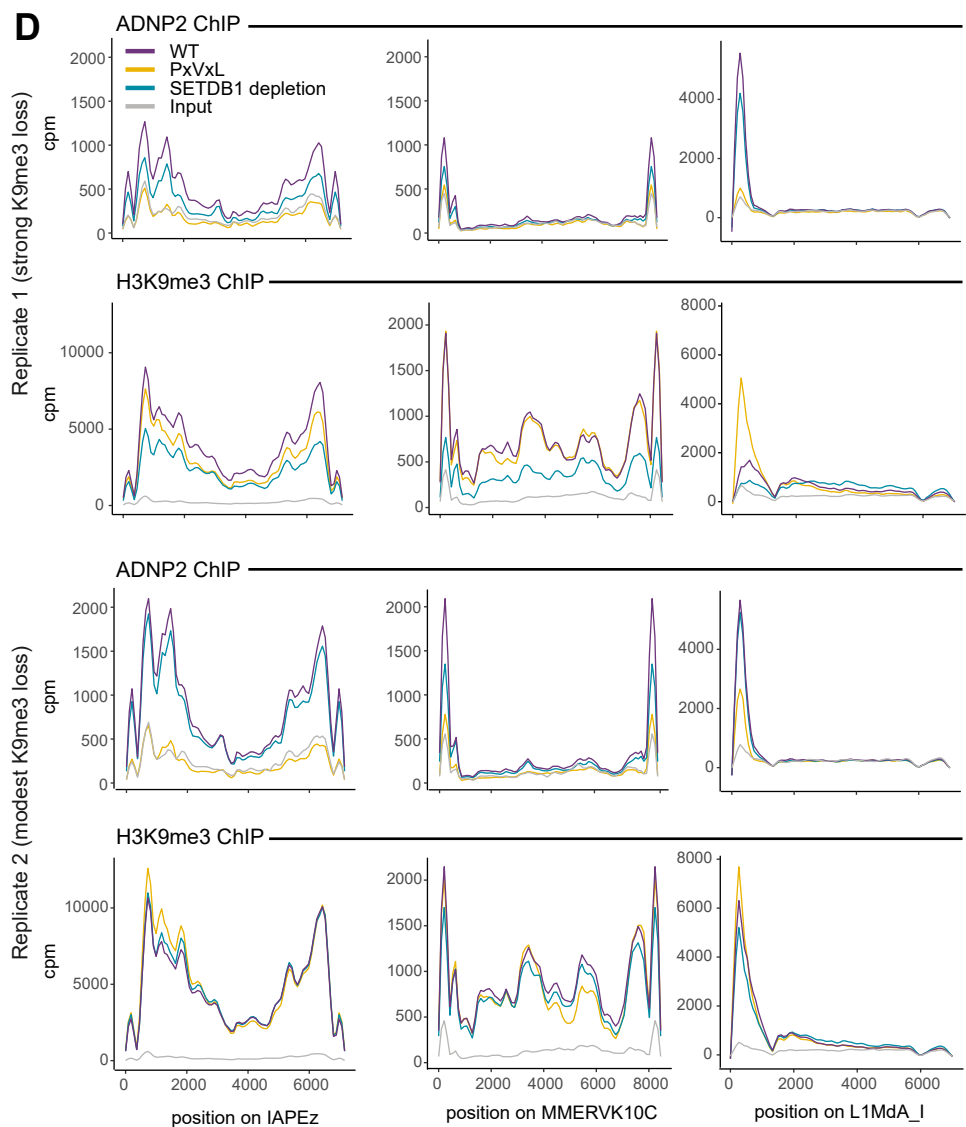

Supplement: Supplement 5 [file Supplemental_FigS5.pdf]

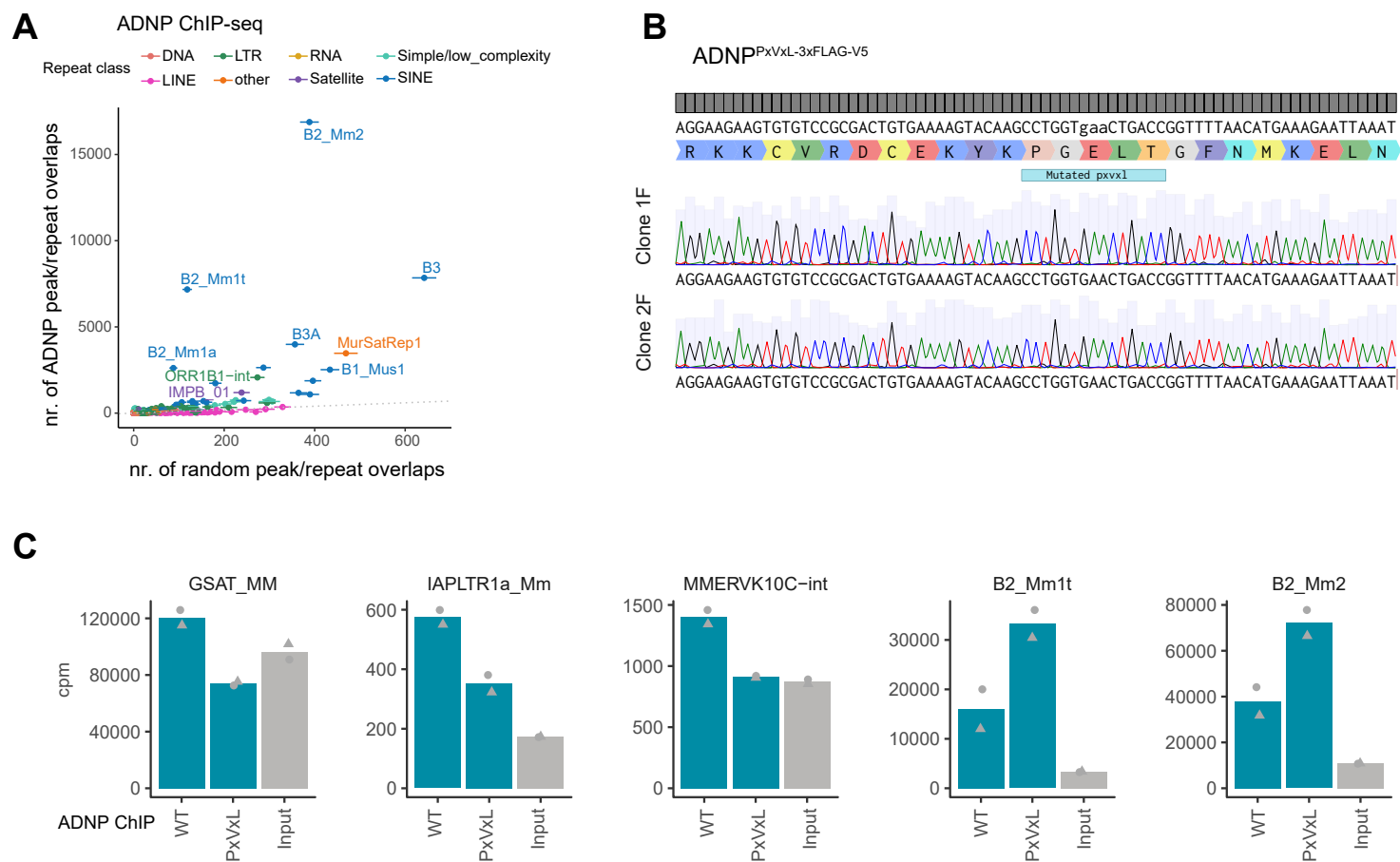

Figure S6

Supplement: Supplement 6 [file Supplemental_FigS6.pdf]

**A**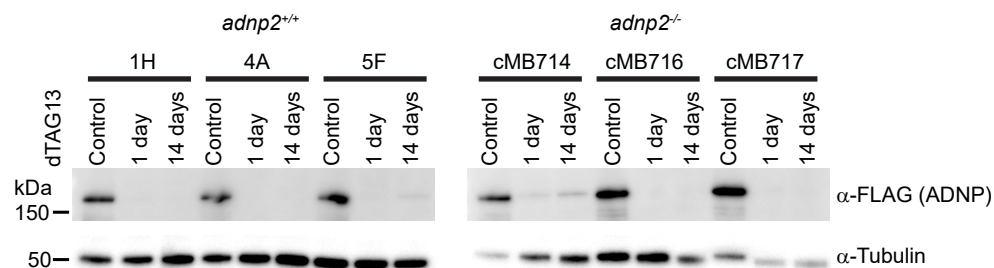**B**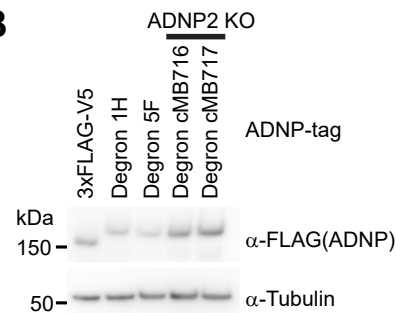**C**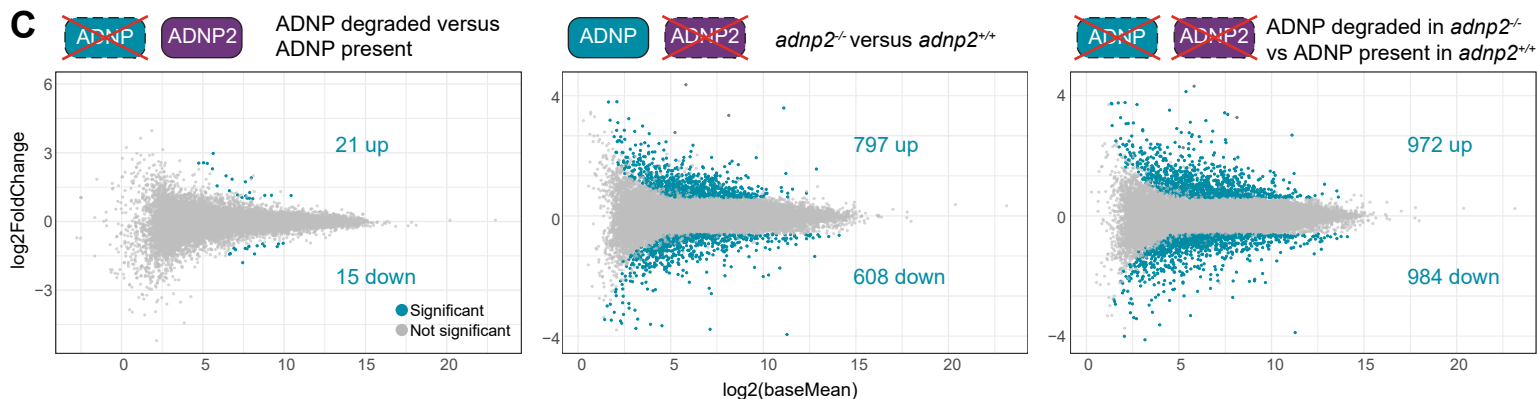**D**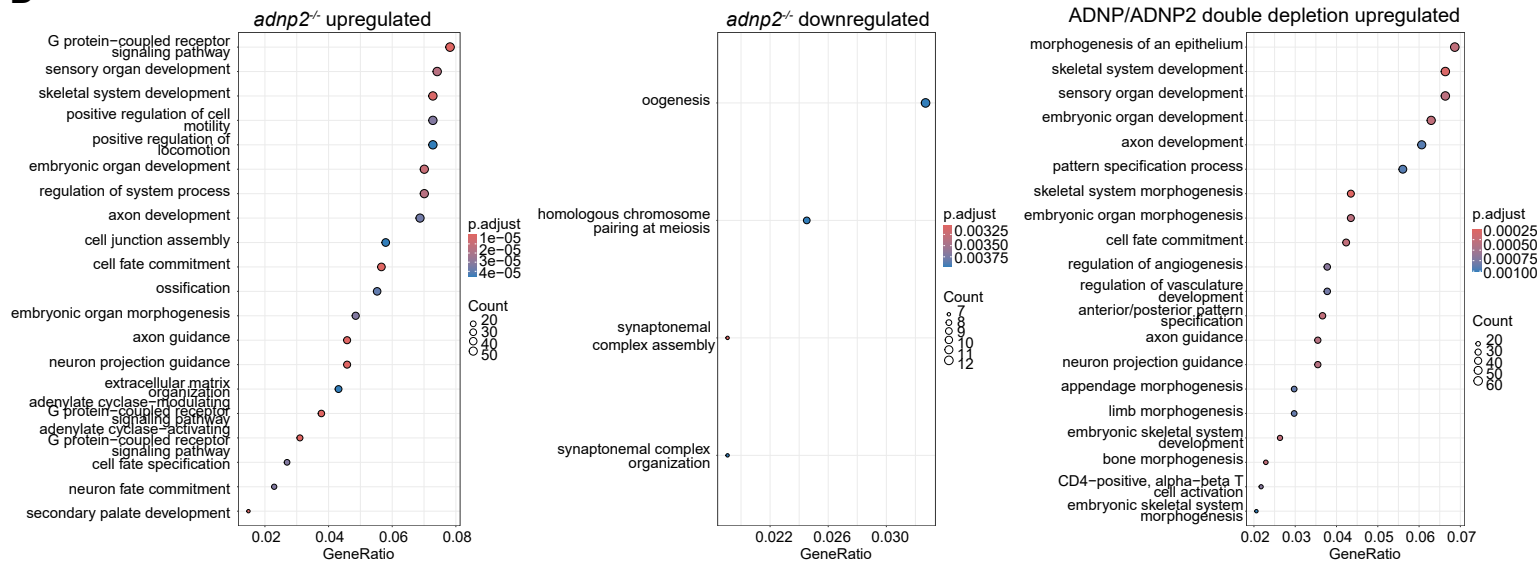**E**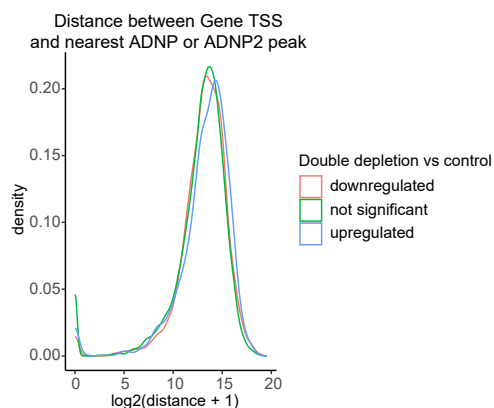

Supplement: Supplement 7 [file Supplemental_FigS7.pdf]

**A**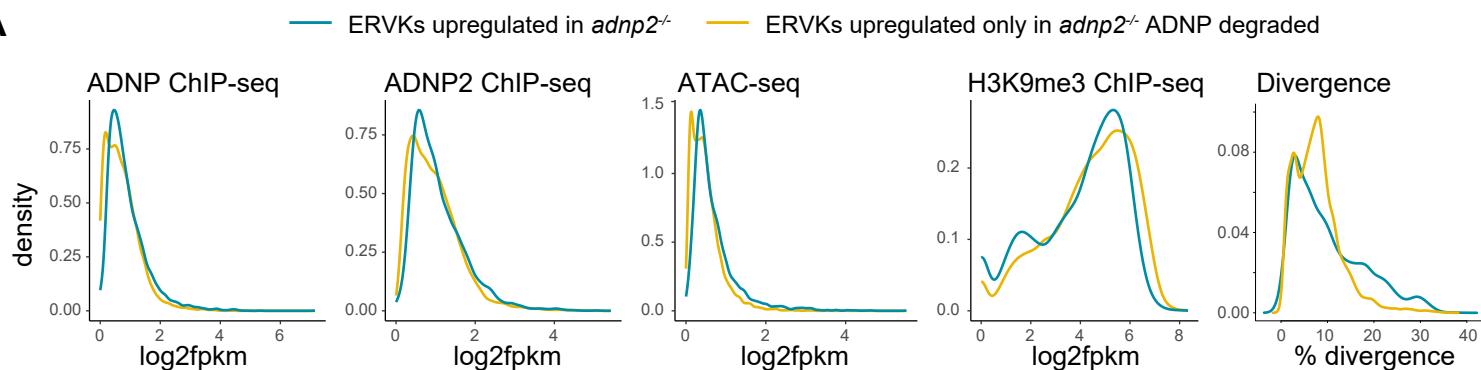**B**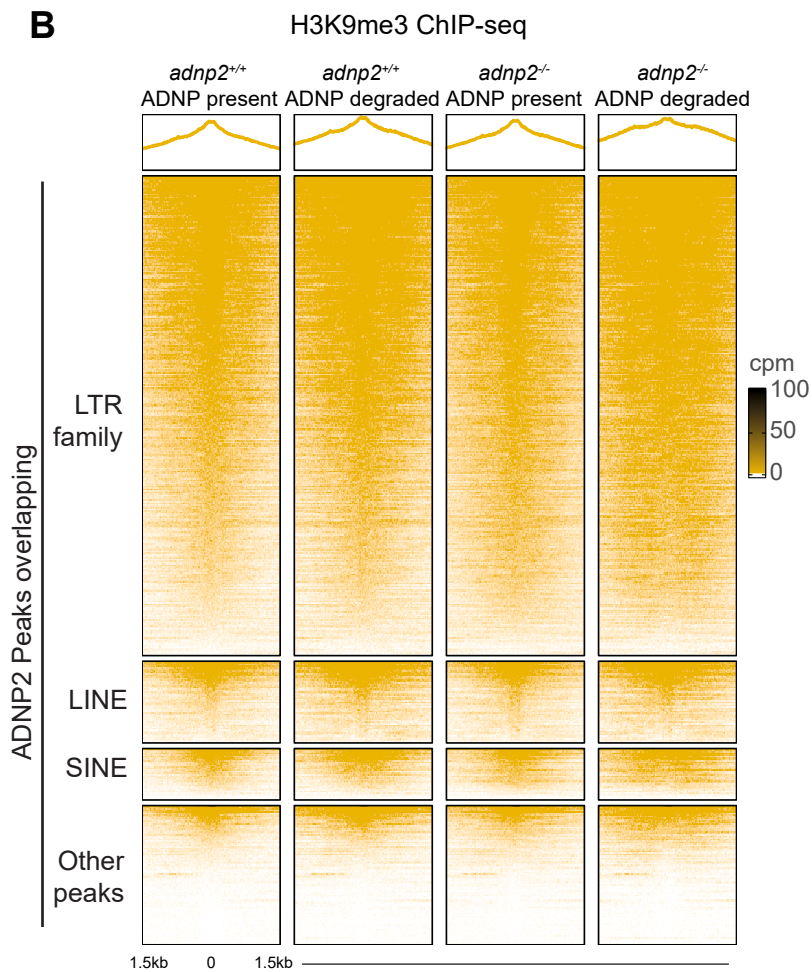**C**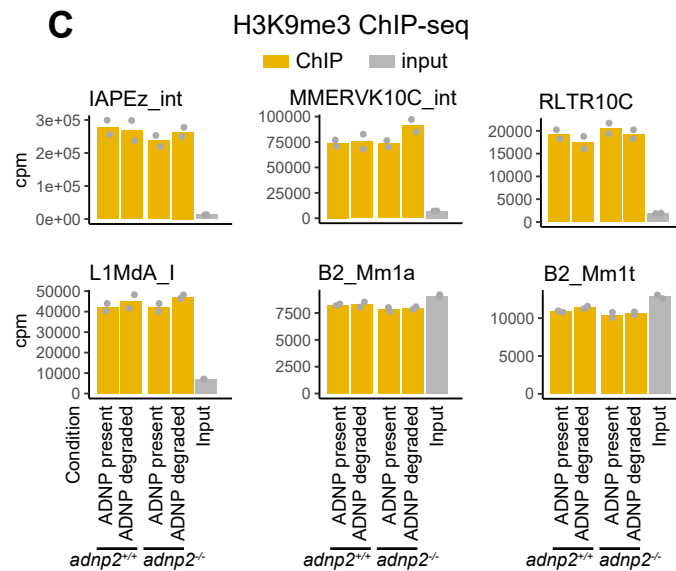**D**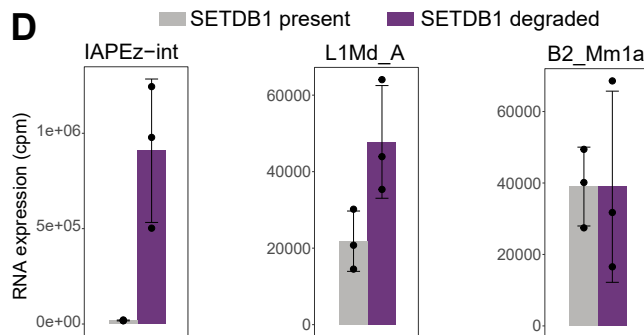

Supplement: Supplement 8 [file Supplemental_FigS8.pdf]
