# Supplementary material for: ChAHP2 and ChAHP control diverse retrotransposons by complementary activities
Source: Genes Dev. 2024 Jun 1;38(11-12):554–68. doi: 10.1101/gad.351769.124 (PMC11293393; doi:10.1101/gad.351769.124)
Supplement: Supplement 9 [file Supplemental_Legends.pdf]

Supplemental Figure Legends

Supplemental Figure S1, related to Figure 1. (A) Predicted ADNP or ADNP2 orthologue sequences from species representing different vertebrate classes were aligned using Clustal omega[60] and visualized with JalView[61]. The conservation scores were scaled and used in Figure 1A. Alignment excerpts for the regions containing Zinc Finger 4 (counting from the N terminus) and PxVxL motif are displayed. The putative region responsible for sequence specificity of the zinc finger is highlighted. **(B)** Sanger sequencing traces of PCR amplicons around the C terminal region of ADNP2 after insertion of the Avi-3xFLAG tag, focused on the site of editing. These cell lines were used in Figure 1B and Figure 1D. **(C)** Sanger sequencing traces of PCR amplicons around the C terminal region of ADNP after insertion of the FKBP-3xFLAG-Avi tag, focused on the site of editing and the STOP codon. These cell lines were used in Figure 1B, Figure 1D and Figure 5. **(D)** RT-qPCR analysis of the indicated RNAs in parental control (cMB581) and derived *adnp*$^{-/-}$ clones. Note that ADNP mRNA is undetectable in the knockout clones under these conditions, and SINE B2 RNA is more abundant. These cell lines were used in Figure 1D **(E)** Normalized RNA sequencing counts for *adnp2*$^{+/+}$ and *adnp2*$^{-/-}$ cells in the ADNP$^{\text{FKBP-3xFLAG-Avi}}$ background, used in Figure 1D and Figure 5.

Supplemental Figure S2, related to Figure 1. (A) ADNP2 or ADNP were deleted using genome editing in endogenously edited lines expressing ADNP$^{\text{FKBP-3xFLAG-Avi}}$ or ADNP2$^{\text{Avi-3xFLAG}}$, respectively, and subjected to immune precipitation against FLAG, followed by proteomics analyses. Comparison of indicated target IP samples as indicated to the untagged controls (n=3). **(B)** Comparison of ADNP IPs between *adnp2*$^{+/+}$ and *adnp2*$^{-/-}$ or ADNP2 IPs between *adnp*$^{+/+}$ and *adnp*$^{-/-}$. C) Intensity values for ChAHP complex components normalised to the bait protein.

Supplemental Figure S3, related to Figure 2. (A) Overlap numbers between repeat annotations and ADNP2 peaks or randomized peakset of equal properties, supporting Figure 2D. Only annotations which overlap with peaks more than 50 times, and with the randomized set less than 200 times are displayed. **(B)** Summed reads over repeat annotations normalized to library size for input and ADNP2 ChIP-seq (mean ± SD, n=4). **(C)** Consensus mapping traces over repeat annotations normalized to library size for input and ADNP2 ChIP-seq (mean ± SD, n=4). The internal consensus sequence was stitched with the 5' end and 3' end consensus sequences to generate the plot. The position of the stitched sequence is highlighted. **(D)** Enriched motifs in ADNP2 ChIP peaks, based on MEME-ChIP. For the top 3 motifs by significance, the motif sequence, position, and representation as a percentage of peaks is shown.

Supplemental Figure S4, related to Figure 3. (A) Example Sanger sequencing trace of a PSVLL-PSELT (PxVxL) mutant cell line in the ADNP2$^{Avi\text{-}3xFLAG}$ background, used in Figure 3. **(B)** Cells expressing endogenously edited ADNP2$^{Avi\text{-}3xFLAG}$ either WT or with a PSVLL-PSELT (PxVxL) mutation were subjected to immune precipitation against FLAG and analyzed by western blotting, with the untagged cell line serving as a control. Inputs and IP excerpts come from the same image, with intervening lanes spliced out. Exposure times are shown for each displayed blot. Asterisk denotes the antibody heavy chain band. **(C)** Cells expressing WT or PxVxL mutated ADNP2$^{Avi\text{-}3xFLAG}$ were analyzed by ChIP sequencing and normalized to human spike-ins. Differentially bound regions identified using edgeR (n=2). **(D)** Overlap between repeat annotations and peaks with significantly decreased ADNP2 binding upon introduction of the PxVxL mutation, or a randomized region set with equal properties (mean ± SD, boostrapped 100 times, NS = not significant). **(E)** Same as (D), but for regions with significantly increased binding. **(F)** Metaplots of H3K9me3-ChIP and ATAC-sequencing reads of the indicated samples centered on ADNP2 peak summits split into

categories based on ADNP2 binding behavior of the PxVxL mutant compared to control. (mean ± SD, n = 2 for H3K9me3, n = 3 for ATAC). **(G)** Same as in (F), but for ADNP2 WT and PxVxL mutant ChIP.

Supplemental Figure S5, related to Figure 3. (A) Example Sanger sequencing traces of the beginning and junction regions in $^{2HA\text{-}FKBP}$SETDB1 cell lines, with an ADNP2$^{Avi\text{-}3xFLAG}$ background (parental = cMB580, Figure S1), used in Figure 3. **(B)** Cells were treated with 500nM dTAG13 for 48h before western blot analysis. **(C)** Correlation of changes in H3K9me3 and ADNP2 ChIP-seq signal over ADNP2 peaks upon depletion of SETDB1. **(D)** Consensus mapping traces over repeat annotations normalized to library size for input and ADNP2 or H3K9me3 ChIP-seq split by replicate. The H3K9me3 attrition is more modest in replicate 2, and reflected in a weaker effect on ADNP2 binding. Sequences were stitched as described in Figure 2E.

Supplemental Figure S6, related to Figure 4. (A) Overlaps numbers between repeat annotations and ADNP peaks or randomized peakset of equal properties (boostrapped 100 times, mean ± SD). **(B)** Sanger sequencing traces of PGVLL-PGELT (PxVxL) mutant cell line in the ADNP$^{3xFLAG\text{-}V5}$ background, used in Figure 4. **(C)** Summed reads over repeat annotations normalized to library size for input and ChIP samples as indicated (mean, n=2, replicates annotated).

Supplemental Figure S7, related to Figure 5. **(A)** Western blot analysis of cell used for the RNA sequencing experiment validating depletion of ADNP one day or 14 days after treatment with 250nM dTAG13 (Control = DMSO). **(B)** Western blot analysis comparing basal levels of ADNP in cell lines with affinity- and degron-tagged ADNP to a line with just an affinity tag. **(C)** Differential

gene expression analysis between ADNP/ADNP2 perturbation and the corresponding unperturbed controls Significantly changing genes are colored (FDR < 0.01, |log2FoldChange| >1, n = 3), **(D)** Biological process GO term enrichment analyses of differentially changing gene sets for the indicated conditions. Note that there were no significantly enriched (FDR < 0.01) terms found for other category/condition combinations. **(E)** Distribution of distances from gene transcription start site to the nearest ADNP or ADNP2 peak split by regulation status in the combined ADNP/ADNP2 removal condition.

Supplemental Figure S8, related to Figure 5. **(A)** Distributions of ADNP ChIP signal intensity, ADNP2 ChIP signal intensity, ATAC-seq signal intensity, H3K9me3 ChIP signal intensity and divergence from consensus for individual ERVK insertions, categorized based on whether they are sensitive to ADNP2 loss (ERVKs upregulated in *adnp*$^{-/-}$) or only significantly upregulated when both ADNP and ADNP2 are removed (ERVKS upregulated only in *adnp2*$^{-/-}$ ADNP degraded) **(B)** Waterfall plot for H3K9me3 ChIPs, centered on ADNP2 peak summits, and split by overlap with select repeat annotations as indicated (mean, n=2). **(C)** Summed reads over repeat annotations normalized to library size for input and a series of ChIP samples as indicated (mean, n=2, replicates represented as points). **(D)** RNA expression normalized to library size for example repeat classes before and after depletion of SETDB1 using 500nM dTAG-13 for 48h (mean ± SD, n=3).
